# The Medicinal Chemistry of Imidazotetrazine Prodrugs

**DOI:** 10.3390/ph7070797

**Published:** 2014-07-10

**Authors:** Catherine L. Moody, Richard T Wheelhouse

**Affiliations:** School of Pharmacy, University of Bradford, Bradford BD7 1DP, UK

**Keywords:** azolotetrazinone, temozolomide, mitozolomide, MGMT, DNA mismatch repair, DNA alkylation

## Abstract

Temozolomide (TMZ) is the standard first line treatment for malignant glioma, reaching “blockbuster” status in 2010, yet it remains the only drug in its class. The main constraints on the clinical effectiveness of TMZ therapy are its requirement for active DNA mismatch repair (MMR) proteins for activity, and inherent resistance through *O6*-methyl guanine-DNA methyl transferase (MGMT) activity. Moreover, acquired resistance, due to MMR mutation, results in aggressive TMZ-resistant tumour regrowth following good initial responses. Much of the attraction in TMZ as a drug lies in its PK/PD properties: it is acid stable and has 100% oral bioavailability; it also has excellent distribution properties, crosses the blood-brain barrier, and there is direct evidence of tumour localisation. This review seeks to unravel some of the mysteries of the imidazotetrazine class of compounds to which TMZ belongs. In addition to an overview of different synthetic strategies, we explore the somewhat unusual chemical reactivity of the imidazotetrazines, probing their mechanisms of reaction, examining which attributes are required for an active drug molecule and reviewing the use of this combined knowledge towards the development of new and improved anti-cancer agents.

1. Introduction7982. Mechanism of Action7993. Elucidation of Prodrug Activation8003.1. The Chemistry of MTZ and ETZ8014. Kinetic Considerations8054.1 Prodrug Activation Kinetics8054.2. Ultimate Electrophile Lifetime8075. Temozolomide Co-crystals8076. Synthesis of Temozolomide and the Imidazotetrazine Core8107. Synthesis of Structural Analogues8177.1. Alternative Cores8177.2. 6- and 8-Analogues8197.3. 3-Analogues8228. Design of MGMT/MMR-Independent Anti-Cancer Agents8258.1. Introduction to NGP Analogues8268.2. Synthesis of Novel N-Linked Imidazotetrazine Dimers8288.3. Properties and Activity of N-Linked Compounds8299. Conclusions931

## 1. Introduction

Imidazotetrazines are a class of bicyclic aromatic heterocycles, exemplified by the DNA methylating agent, temozolomide (Temodar^®^, Temodal^®^, TMZ, **1a**). Notable previous reviews of the imidazotetrazines have focused on the development of TMZ [1,2] and the clinical properties and significance of TMZ [3,4]. The last detailed analysis of imidazotetrazine chemistry appeared in 1990 [5]. TMZ entered clinical trials in 1985 and since its release to the market by Schering-Plough in 1999 [6], has been widely used in combination with radiotherapy as the standard first-line treatment for malignant glioma (glioblastoma multiforme, grade IV astrocytoma). Somewhat serendipitously, as a drug it has excellent features: it is a stable solid which is also acid stable, allowing for 100% oral bioavailability, so administration is straightforward in comparison with many anticancer drugs. Absorption is rapid, with peak plasma concentration achieved within 0.33–2 h. Biodistribution is excellent (Vd 17 Lm^−2^) [7], and PET studies using ^11^C-labelled prodrug have provided direct evidence of blood-brain barrier penetration [8]. In 2010, TMZ reached blockbuster drug status, yet despite this impressive record it remains the only member of its class in the clinic. In this review we will explore the reasons for this and what makes TMZ special, along with surveying current efforts to achieve new effective drugs in this class.

The early history of imidazotetrazinone pharmacology indicated a considerable activity cliff with only a very small number of analogues exhibiting anticancer activity *in vitro* or *in vivo*. The 3-haloethyl analogues had good activity, the 3-methyl was of moderate effectiveness but other analogues, as exemplified by the 3-ethyl, were inactive (Table 1) [9]. The solution to this conundrum and the vision to design active new analogues arose from detailed definition of the mechanism of prodrug activation using kinetic NMR experiments. Indeed, TMZ entered clinical trials before the final details of prodrug activation chemistry were elucidated.

**Table 1:** Activity of various TMZ analogues against TLX5 lymphoma implanted s.c. in CBA/CA mice. Adapted with permission from reference [9]. Copyright (1987) American Association for Cancer Research.

	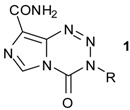		
	**R**	**Dose mg/kg/day**	**T/C *^b^* %**
**1a**	CH_3_ (TMZ)	160	151
		80 *^a^*	154
		40 *^a^*	181
**1b**	(CH_2_)_2_Cl (MTZ)	40	458
		16 *^a^*	302
**1c**	CH_2_CH_3 _(ETZ)	640	123
		80 *^a^*	111
**1d**	(CH_2_)_2_Br	160	137
**1e**	(CH_2_)_2_CH_3_	320	103
**1f**	(CH_2_)_2_OCH_3_	320	98
**1g**	(CH_2_)_3_Cl	320	108
**1h**	CH_2_CHClCH_2_Cl	320	103
**1j**	CH_2_CH=CH_2_	320	97
**1k**	CH(CH_3_)CH_2_CH_3_	320	83
**1m**	(CH_2_)_5_CH_3_	320	103

*^a^* Divided dose schedules; *^b^* treated *versus* control.

## 2. Mechanism of Action

**Scheme 1:**
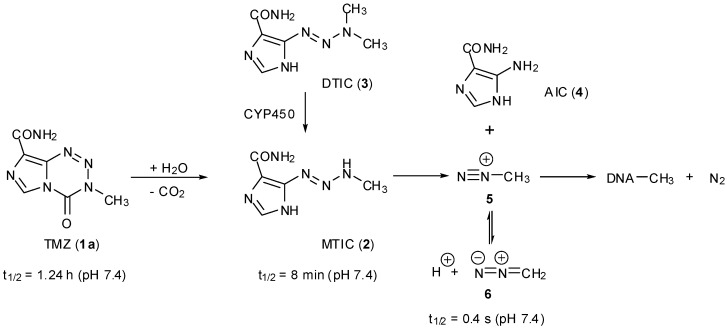
Prodrug activation of TMZ.

TMZ (**1a**) is in fact a prodrug of a prodrug and its aqueous chemistry is typical of imidazotetrazine compounds (Scheme 1). Under neutral or alkaline conditions it undergoes hydrolytic ring opening, under purely chemical control, to give the open chain triazene MTIC (**2**) as the first significant intermediate. The prodrug dacarbazine (DTIC, **3**), which is used to treat a number of cancers including malignant melanoma, also shares this intermediate but in contrast requires hepatic demethylation via CYP450 oxidation and loss of formaldehyde to release MTIC [10]. This activated intermediate fragments to liberate AIC (**4**) and methyl diazonium ions **5** which then react with nucleophilic sites on DNA; it is the interaction of this methylated DNA with various DNA repair pathways that elicits the cell killing response.

Methylation of DNA occurs at a number of nucleophilic sites, the majority of which appears at guanine-*N*7 (70%) with a further 9% at adenine-*N*3 [1]. This *N*-methylation, however, is of no therapeutic value since the lesions are efficiently handled by base excision repair (BER) enzymes [11]; the anti-cancer activity of TMZ is solely attributed to the small fraction (5%) of methylation which occurs at guanine-*O*6.

The therapeutic effect of TMZ is dependent on the presence of DNA mismatch repair (MMR) proteins. *O*6-Methylguanine results in a mispairing during replication as it preferentially forms a wobble base pair with thymine. This is recognised by MMR which triggers a response; that is, T is identified, removed but then replaced with another T. Futile cycles of excision/repair result in depletion of dTTP, ultimately leading to long-lived strand breaks and cell death [1,11,12]. The G*O*6 methyl adduct can, however, be repaired by *O*6-methylguanine-DNA methyltransferase (MGMT), resulting in active resistance to TMZ in tumours in which MGMT is proficient. Acquired resistance to TMZ can be brought about by mutations in MMR, in particular the MSH6 subunit that recognises G*O*6 modifications, or enhanced MGMT activity through promoter hypomethylation [13]. The action of MGMT is not enzymatic; the protein is consumed stoichiometrically during the repair process and as such, inactivation of the protein can have a beneficial effect. With tumour cells high in MGMT levels, pre-treatment with inactivators such as *O*-benzylguanine has been shown to enhance the *in vitro* and *in vivo* activity of TMZ [14]. Several combination studies with inhibitors of alternative repair processes have also been carried out and are reviewed elsewhere [11].

It is evident that in order for a tumour to be responsive to TMZ treatment, it must be deficient in MGMT whilst also proficient in MMR; absence of the latter results in a tolerant phenotype whilst presence of MGMT impedes therapy regardless of MMR status.

## 3. Elucidation of Prodrug Activation

**Figure 1:**
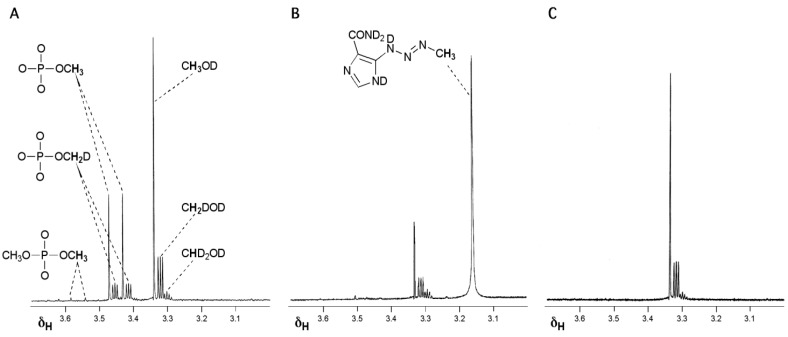
Reaction of TMZ, MTIC and CH_2_N_2_ in D_2_O systems. (**A**) Products of reaction of TMZ with phosphate buffer pD = 7.8 showing deuterium incorporation into methanol and methyl phosphate; (**B**) MTIC in Na_2_CO_3_ (10% in D_2_O) showing the intact isotopic configuration of the MTIC methyl and deuterium incorporation into the methanol product of hydrolysis; (**C**) Products of condensing diazomethane into Na_2_CO_3_ (10% in D_2_O) [15,16,17].

The key experiment attempted to identify intermediates in the prodrug activation pathway by time-dependent NMR spectroscopy [15,16,17]. Although the experiment was too insensitive to detect any reactive intermediates directly, the NMR data proved crucial to understanding the prodrug activation mechanism. When TMZ was reacted in a phosphate buffer/D_2_O system at pD = 7.8, designed to mimic physiological pH, the methyl group bearing products (methanol and methyl phosphate) showed significant loss of ^1^H signal and the emergence of complex multiplet signals typical of ^1^H–^2^H geminal coupling in the products (Figure 1A). It appeared that during the reaction, all three ^1^Hs of the TMZ methyl became freely exchangeable with the solvent so that the ^1^H/^2^H ratio in the final products of methyl group transfer reflected the relative proportions of ^1^H and ^2^H in the H_2_O/D_2_O solvent mixture. Furthermore, at high pH it proved possible to simultaneously observe MTIC (**2**) which did not exhibit methyl group ^1^H/^2^H exchange as well as its reaction products where similar ^1^H/^2^H exchange was evident (Figure 1B). The final experiment to confirm the diazonium intermediate involved condensing diazomethane (**6**) into the H_2_O/D_2_O systems whereupon the products of ^1^H/^2^H exchange again appeared (Figure 1C); indeed this process was similar to the published synthesis of isotopically-labelled diazomethane [18]. These experiments established the position of methyldiazonium as the ultimate electrophile in the prodrug activation mechanism of TMZ (Scheme 1).

### 3.1. The Chemistry of MTZ and ETZ

**Figure 2:**
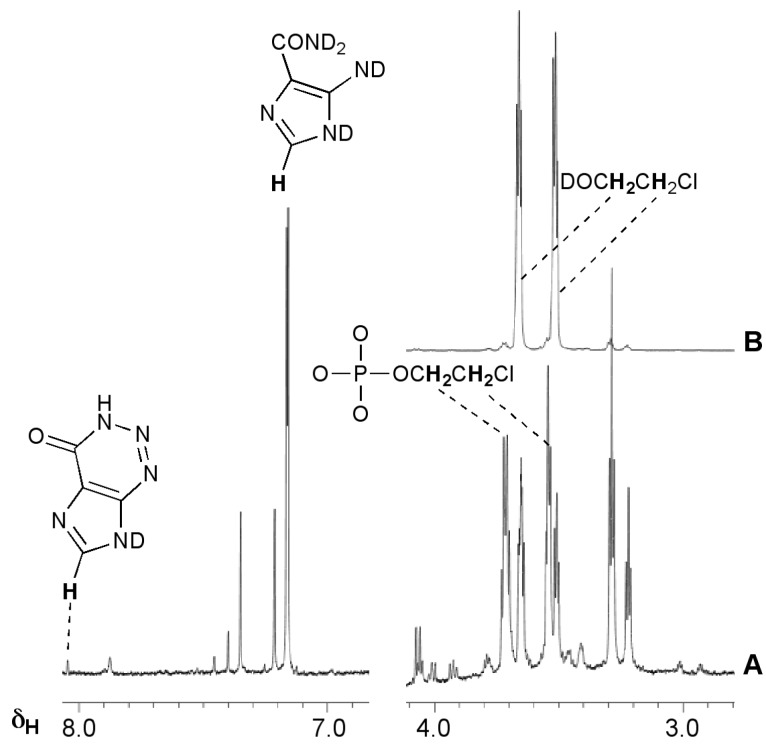
Reaction of MTZ with phosphate buffer pD = 7.8, 37 °C. (**A**) ^1^H-NMR spectrum after 14 days; (**B**) The same sample spiked with authentic chloroethanol (**7**).

**Scheme 2:**
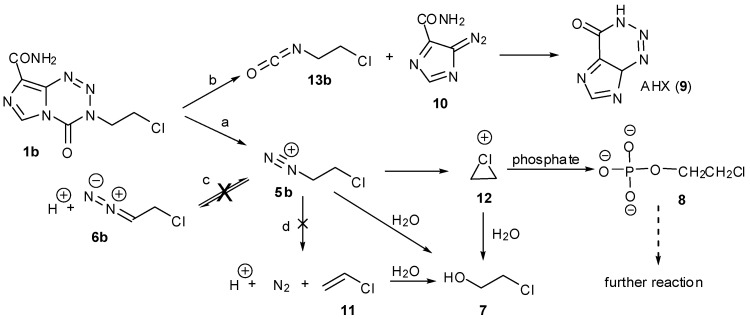
The aqueous chemistry of MTZ.

The realisation that diazonium ions were formed by triazenes allowed explanation of the activity and inactivity of other imidazotetrazine analogues. MTZ as the more potent (but myelosuppressive), first-generation imidazotetrazine prodrug was shown to be activated predominantly through the chloroethyltriazene analogue of MTIC [19,20]. The chloroethyldiazonium ions **5b** that would be derived this way have a complex aqueous chemistry involving elimination and hydrolysis pathways that was extensively investigated for reactive intermediates generated by the chemically-related diazonium prodrugs, the chloroethyl nitrosoureas [21]. NMR experiments with MTZ confirmed the complexity of this chemistry in the context of the imidazotetrazines (Figure 2, Scheme 2). The ^1^H-NMR spectra were more complex than for TMZ and the reaction took longer to reach completion. As expected, chloroethanol (**7**) and alkylated phosphates **8** were the principal aliphatic products; in the aromatic region, the major product was AIC (**4**) with a trace of 2-azahypoxanthine (AHX, **9**), the product of intramolecular trapping of the TMZ precursor diazo-IC **10**, and at least two other modified imidazoles. In contrast to TMZ, there was no evidence of deuterium incorporation into any products (Figure 2, Scheme 2c), neither was there evidence of elimination to vinyl chloride **11** (Scheme 2d). These observations are significant as they suggest a role for the chlorine atom in diazonium ion stability, for example by formation of a full or partial chloronium ion **12**. Other DNA reactivity considerations also point to the involvement of a chloronium intermediate [22].

Interestingly, reaction in lower alcohols and acetonitrile at elevated temperatures followed a retro-cycloaddition route, as evidenced by detection of degradation products of diazo-IC, *i.e.*, AHX, and chloroethyl isocyanate (**13b**) (Scheme 2b). The trace of AHX produced in the aqueous buffer system showed that this route, although not preferred, exists in aqueous systems, giving credence to early reports of MTZ as a carbamoylating agent [23]. Incidentally, the trifluoroethyl analogue only reacted by the retro-cycloaddition route in aqueous systems (*vide infra*, Scheme 4).

**Figure 3:**
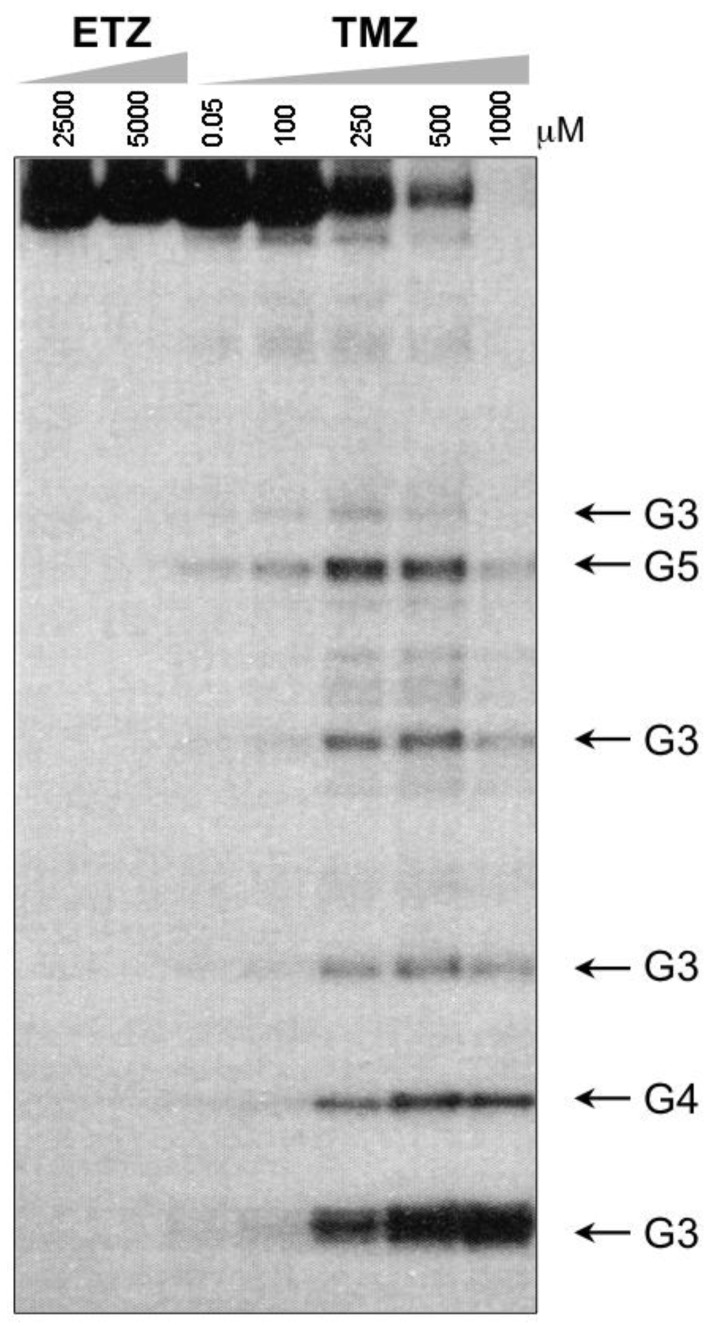
Comparison of reaction of ETZ and TMZ with DNA, showing the unreactivity of ETZ up to 5 mM, sites of G*N*7 alkylation produced in the 276-bpBamHI-Sal1 fragment of pBR322, by the piperidine cleavage method. Adapted from reference [24].

**Scheme 3:**
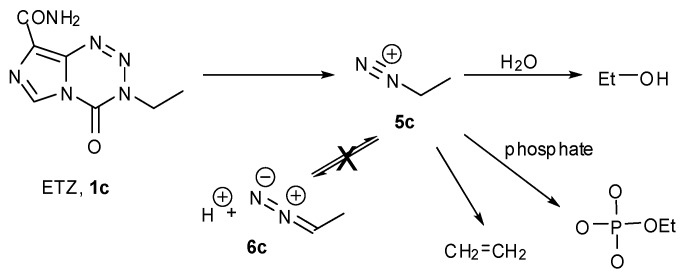
The aqueous chemistry of ETZ.

A great anomaly of the early compounds was the inactivity of the ethyl and higher alkyl analogues (*vide supra*, Table 1). In reaction with DNA it was impossible to detect G*N*7 alkylation sites, even at very high dose (5 mM) of ethazolastone (ETZ, **1c**) and there was no *in vitro* or *in vivo* antitumour activity (Figure 3) [24]. The reason for this becomes apparent when the fates of ethyldiazonium ions are considered (Scheme 3) [25].

**Figure 4:**
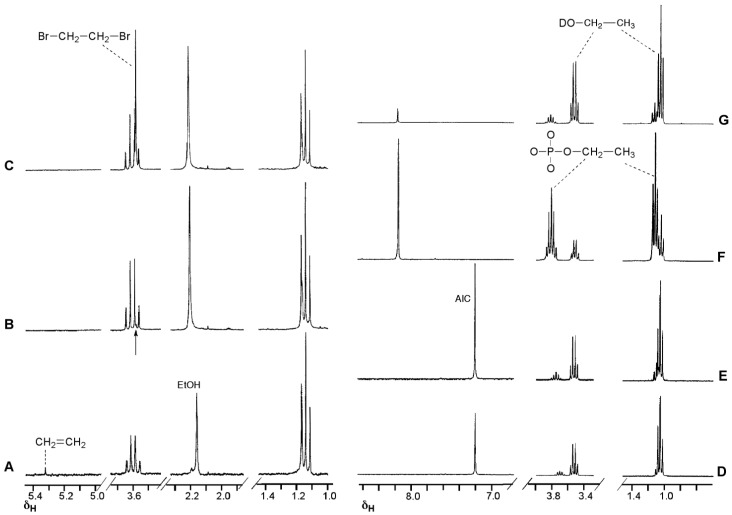
^1^H-NMR investigation of the reaction of ETZ with phosphate buffer (pH 7.8) in a two phase system (D_2_O/CDCl_3_). Spectra A–C CDCl_3_ layer, spectra D–G, D_2_O layer. (**A**) At the end of the imidazotetrazine hydrolysis reaction identifying the putative ethene peak; (**B**) after addition of Br_2_, the arrow indicates the position of a new peak; (**C**) sample of spectrum B after adding authentic dibromoethane; (**D**) at the end of the imidazotetrazine hydrolysis reaction; (**E**–**G**) sample of spectrum D after sequentially spiking with authentic AIC, ethylphosphate and ethanol (note the shift in the AIC signal after adding the acidic ethyl phosphate).

The NMR experiment in deuterated buffer was relatively uncomplicated (Figure 4). In this case the experiment was conducted in a two phase D_2_O/CDCl_3_ system. Deuterium incorporation was not detected in any products so, as with MTZ, the diazoalkane equilibration with **6c** did not occur. Ethanol and ethylphosphate were confirmed as the major reaction products in the D_2_O layer. In the CDCl_3_ layer, a singlet peak appeared at 5.3 ppm in the ^1^H spectrum. Reaction with Br_2_ and subsequent spiking with authentic dibromoethane identified the by-product as ethene: a result of elimination of ethyldiazonium **5c**. This implies that the fate of ethyldiazonium is either immediate nucleophilic substitution or rapid elimination to ethene without time for diazoethane equilibration. In the former case, the incipient electrophile may be too short lived to effectively locate a reaction site on DNA, while the latter route results in drug inactivation by elimination.

**Scheme 4:**
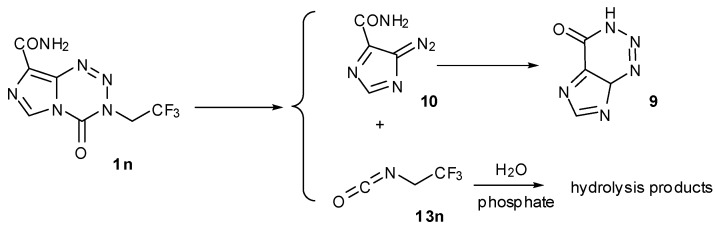
The aqueous fate of the 3-trifluoroethylanalogue **1n**.

The need to block elimination led first to analogues lacking hydrogen atoms in the β-position of the side chain such as the 3-furfuryl and 3-trifluoroethyl **1n** (Scheme 4) [25]. Of these, the trifluoroethyl analogue showed reasonable activity in the NCI60 cell line panel, with mean GI_50_ = 10.7 µM (log range = 2.35) compared with MTZ (79 µM, log range = 0.96), with a distinctly different pharmacological profile from MTZ (*p* = −0.347). Interestingly, a sample dissolved in DMSO showed clean retro-cycloaddition to give AHX and trifluoroethylcarbamic acid on standing overnight at room temperature. In a capricious turn of fate, investigation of the aqueous prodrug activation mechanism by ^1^H-, ^31^P- and ^19^F-NMR showed a complex mixture of products that included AHX and a mixture of at least nine fluorine containing compounds of which trifluoroethanol and trifluoroethylamine were components [25]. The aliphatic portion of the product mixture spectra (^1^H and ^19^F) could be replicated by reaction of trifluoroethylisocyanate (**13n**) with buffer. This is a contrast to MTZ where heating in a non-aqueous system was required to effect the retro-cycloaddition and indicates that the balance between retro-cycloaddition and hydrolysis at *C*4 is determined by the electron withdrawing effect of the 3-substituent.

## 4. Kinetic Considerations

The data in Scheme 1 highlight two kinetic parameters key to the design of new agents. The first is the rate of addition of water (or hydroxide ion) to initiate the ring-opening reaction. The prodrug itself must be sufficiently stable to allow uptake and distribution without significant hydrolysis. The second is the reactivity of the latent electrophile.

### 4.1. Prodrug Activation Kinetics

**Figure 5:**
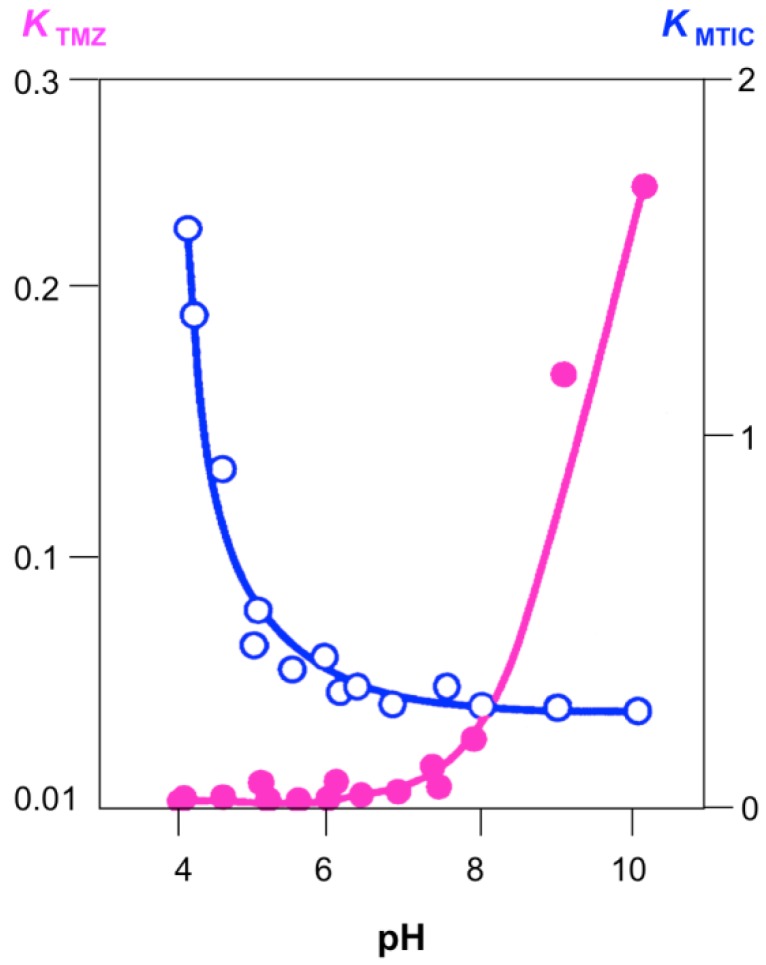
pH Dependence of the pseudo-first order rate constants for hydrolysis of TMZ and MTIC. Adapted from reference [16].

An important property of the imidazotetrazines is the contrast of robust acid stability with rapid alkaline hydrolysis (Figure 5); the slow hydrolysis at low pH confers acid stability and the convenience of oral dosing [16]. The pH-dependence of this reaction also influences distribution of the prodrug around the body: hydrolysis kinetics at pH 7.4 match closely the uptake rate (peak plasma concentration after 30 min) and metabolic half-life (t_½_ = 1.29 h) in patients [1,9]. If the aqueous lifetime were too short, the prodrug would be inactivated long before it were able to reach its sub-cellular target; too long it could be metabolised and excreted without taking effect. The pH dependence of TMZ and MTIC hydrolysis are complementary, so that it is only around neutral pH that the whole reaction process from prodrug activation to methyl group transfer occurs effectively, indeed MTIC and similar triazenes may be prepared by alkaline hydrolysis of their imidazotetrazines [20].

**Figure 6:**
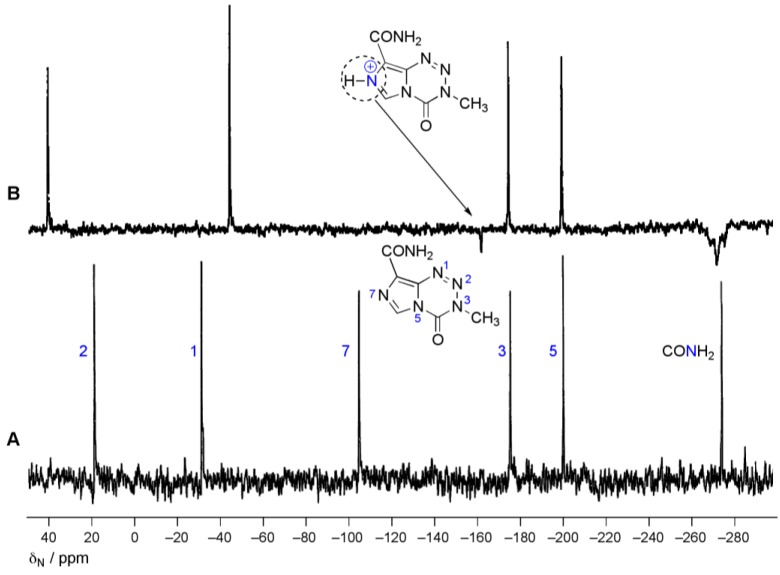
^15^N-NMR spectra of TMZ. (**A**) ^15^N{^1^H} in DMSO-*d_6_*; (**B**) with full ^1^H nOe in TFA. Both experiments were acquired in the presence of Cr(acac)_3_ and referenced to external CH_3_NO_2_ [26].

Superficially, imidazotetrazine activation is hydrolysis of a carbonyl compound with water or hydroxide ion attacking at *C*4, so that both acid and base catalysed reactions would be expected, in common with other carbonyl group reactions. The reason for the absence of the acid catalysed variant is the presence of the weakly basic imidazole *N*7. ^15^N-NMR experiments showed that the resonance of *N*7 was shifted and inverted in the presence of TFA, indicating that it carried a proton and was also, formally, the site of the positive charge (Figure 6) [26]. Since protonation occurs at a distant site, it is not able to activate the 4-carbonyl group to nucleophilic attack. The site of protonation at *N*7 was independently determined in the X-ray crystal structure of the salt TMZ·HCl·2H_2_O (*vide infra*, Figure 9) [27].

In addition to pH, the aqueous reactivity of imidazotetrazines generally is also dependent on the nature of the 3-substituent, such that the rate of hydrolysis is increased by electron withdrawing groups [20], notwithstanding the ability of powerfully electron withdrawing groups to switch the mechanism away from hydrolysis to the retro-cycloaddition reaction.

### 4.2. Ultimate Electrophile Lifetime

The other significant kinetic parameter is the lifetime of the ultimate electrophile released from the prodrug. Hydrolysis of methyldiazonium in a purely chemical system has t_½_ = 0.39 s [28]. Again, a sub-optimal value detracts from clinical effectiveness: ethyldiazonium eliminates or reacts with water before it has time to encounter a reactive nucleic acid target site [25], while longer-lived intermediary electrophiles such as chloronium achieve clinical efficacy [29]. For TMZ, these clinically-useful, if not formally optimized, pharmacokinetic properties were achieved serendipitously.

## 5. Temozolomide Co-Crystals

Drug stability, solubility and formulation are important factors when considering activity *in vivo*, as they can be barriers to bioavailability and efficacy. These factors are influenced by the solid form in which the compound of interest resides, whether this be as polymorphs, salts, solvates, co-crystals or even incorporated into microspheres [30].

**Figure 7:**
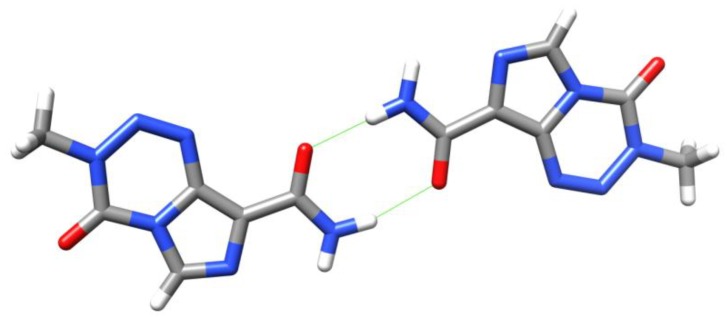
Dimers seen in Lowe’s crystal structure of TMZ [33]. Strong H-bonds are shown in green.

Drug compounds can frequently exist in a number of different polymorphs. Ten polymorphs of TMZ are covered in the patent literature [31,32], of which four have had X-ray structures solved [33,34]. TMZ can easily form a dimer via intermolecular hydrogen bonds between the carboxamide groups (Figure 7). It has also been proposed that the energetic preference for the observed conformer can be attributed to an intramolecular hydrogen bonding interaction between amide NH and imidazole *N*7, although this shows significant (74°) deviation from linearity [27,33].

Stored under the correct conditions, commercial TMZ is relatively stable. However, prolonged storage or exposure to humid conditions can accelerate degradation of the prodrug and reduce efficacy. Salt formation and crystallisation are widely accepted methods of altering physicochemical properties of drug molecules.

**Figure 8:**
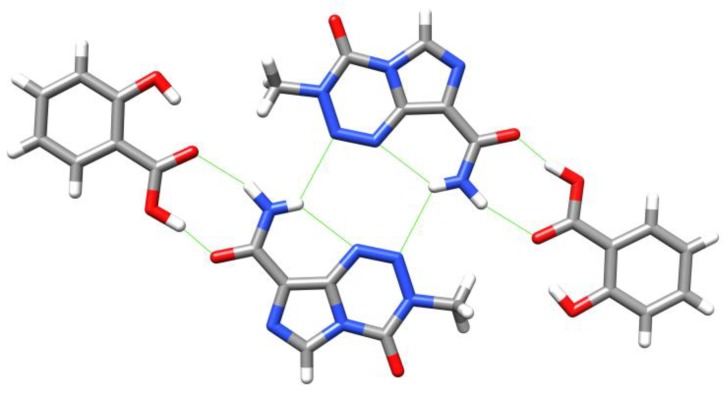
Temozolomide–salicylic acid co-crystal [35]. Strong H-bonds are shown in green.

In recent years, a great deal of work has been done, primarily by Babu and co-workers, to investigate the co-crystalisation of TMZ with different molecules as a means of enhancing stability and solubility. Given the higher stability of TMZ under acidic conditions, Babu *et al*. reasoned that co-crystallisation with compounds bearing generally-recognised-as-safe (GRAS) carboxylic acid groups could act as pH adjusters to inhibit hydrolytic degradation and thus improve aqueous stability. Recognising that complexation must be carefully controlled to ensure that the active species is still released, they made a number of co-crystals with organic acids which were characterised by powder X-ray diffraction and IR before testing (for an example, see Figure 8). As expected, these co-crystals all exhibited a stable hydrogen bonding interaction between the acidic group of the co-former and the amide group of TMZ. Several co-crystals showed dissolution rates comparable to that of TMZ and they all showed a decrease in decomposition rate in pH 7.0 buffer, with a moderate correlation between drug half-life and acidity of the co-former. Whereas TMZ would exhibit discolouration after just one week at ambient conditions, co-crystals with succinic acid, oxalic acid or salicylic acid showed no sign of discolouration or degradation after one year under the same conditions [35].

In a similar vein, Sanphiu, Babu and Nangia have also created a number of co-crystals using GRAS carboxamides such as saccharin and caffeine as co-formers [36]. These two co-crystals were more stable than other carboxamides used, and indeed temozolomide; in the case of saccharin this can be attributed to its acidic nature. However, the stability improvement was less than that seen with carboxylic acid co-formers.

**Figure 9:**
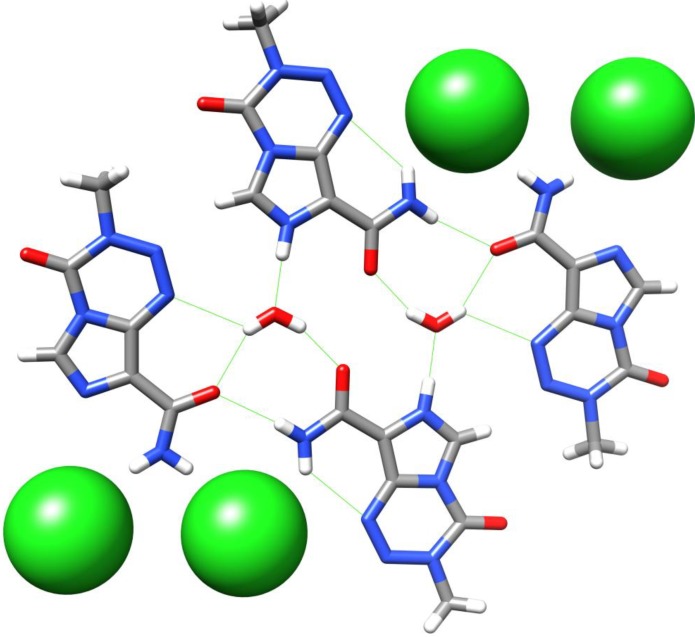
A tetrameric unit of TMZ in the crystal structure of TMZ·HCl·2H_2_O [27]. Strong H-bonds are shown in green.

Babu *et al*. have recently obtained a crystal structure of TMZ hydrochloride in its dihydrate form. It is shown that the lattice structure for this crystallisation form contains one neutral TMZ, one TMZ·HCl, one H_3_OCl and three water molecules, with protonation, as expected from previous NMR studies [26], occurring at the imidazole *N*7 of TMZ (Figure 9). The network of hydrogen bonds forms a tetrameric TMZ unit which extends into parallel sets of ribbons, connected by strong hydrogen bonds to water and chloride ions [27]. Under accelerated humidity conditions, after one week the PXRD plot of the hydrochloride salt had reverted to that of neutral TMZ.

**Figure 10:**
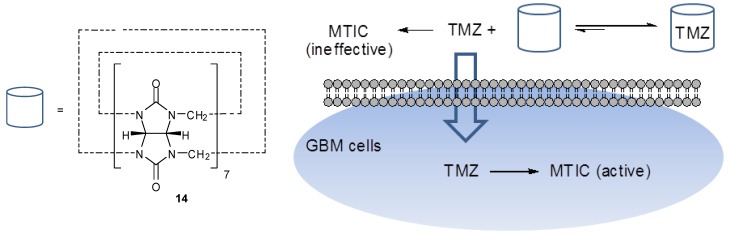
Cucurbit[*n*]uril (**14**) and uptake of TMZ into GBM cells [38].

Other TMZ complexes include that with cadmium chloride, synthesised by Liu [37]. Appel *et al*. exploited the use cucurbit[*n*]uril (**14**), which has previously been shown to improve the solubilty, stability and cellular uptake of drug molecules. They formed a 1:1 complex with TMZ, encapsulating the drug within the natural cavity of the macrocycle (Figure 10). In doing so, the stability of the imidazotetrazine was increased, resulting in a longer half-life (5 h in pH 7.0 buffer, compared with 3 h for the control solution of TMZ alone). It was observed that the activity of the drug against glioblastoma multiforme (GBM) primary cells was increased, presumably due to the prolonged lifetime of TMZ in solution. This may allow increased absorption into cells prior to hydrolysis and thus increased exposure to the active drug since MTIC itself does not cross the blood-brain barrier or effectively penetrate cells [38].

Other TMZ complexes include that with cadmium chloride, synthesised by Liu [37]. Appel*et al*. exploited the use cucurbit[*n*]uril (**14**), which has previously been shown to improve the solubilty, stability and cellular uptake of drug molecules. They formed a 1:1 complex with TMZ, encapsulating the drug within the natural cavity of the macrocycle (Figure 10). In doing so, the stability of the imidazotetrazine was increased, resulting in a longer half-life (5 h in pH 7.0 buffer, compared with 3 h for the control solution of TMZ alone). It was observed that the activity of the drug against glioblastoma multiforme (GBM) primary cells was increased, presumably due to the prolonged lifetime of TMZ in solution. This may allow increased absorption into cells prior to hydrolysis and thus increased exposure to the active drug since MTIC itself does not cross the blood-brain barrier or effectively penetrate cells [38].

## 6. Synthesis of Temozolomide and the Imidazotetrazine Core

**Scheme 5:**
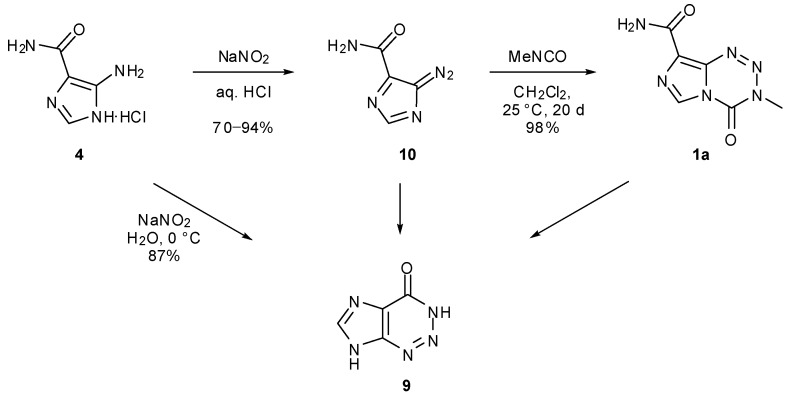
Initial synthesis of TMZ from AIC∙HCl and routes to AHX [20,25,26,40,41,42].

The original synthetic strategy towards TMZ and other imidazotetrazines is based on a general method for azolotetrazines [39], and involves the coupling of 5-diazoimidazole-4-carboxamide (diazo-IC, **10**) or its analogues with the relevant isocyanate (Scheme 5). Evidence suggests this occurs in a stepwise rather than a concerted mechanism [20,25]. Diazo-IC can be readily prepared on a multigram scale from commercially available AIC hydrochloride **4** by treatment under diazotisation conditions (NaNO_2_/HCl), which produces the desired product in yields of 70%–94% [26,40]. Incidentally, if acidic conditions are not used in this reaction, AHX is instead furnished as the sole product [41]. Cyclisation of diazo-IC to AHX occurs rapidly under basic conditions but also takes place more slowly in neutral and acidic media, doing so more readily when light is present [40,42]. In addition, AHX has been observed as result of imidazotetrazine decomposition [20,25].

Early examples of the coupling of diazo-IC with isocyanates were performed in dichloromethane or ethyl acetate, keeping the reaction in the dark to avoid UV-promoted decomposition of diazo-IC. Although use of these reaction solvents results in clinical grade material, reaction times are very slow due to the insolubility of diazo-IC, typically taking several weeks to reach completion [20]. More recent synthetic protocols generally employ DMSO as the reaction solvent, giving rise to much faster reaction times [26,43]. Attempts using a range of imidazolonium and phosphonium ionic liquids failed to improve on the rate, yield or purity of the DMSO reaction [44]. A mixed DMSO–EtOAc solvent has also been used to give large amounts of clinical grade TMZ in three days [45]. Isolation of the imidazotetrazine products can often be achieved by addition of water or ethyl acetate and collection of the resultant precipitate.

**Scheme 6:**
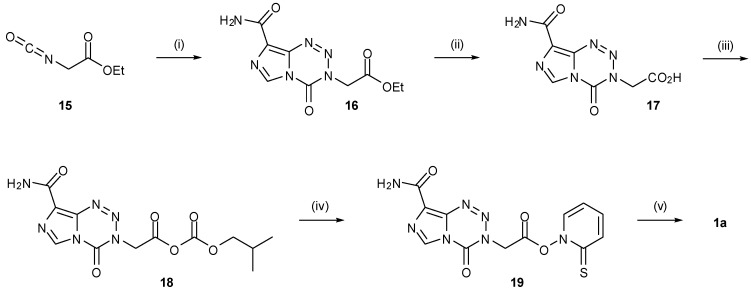
Masked methyl isocyanate approach to TMZ [46].

The stigma associated with the use of methyl isocyanate, following the 1984 Bhopal disaster and subsequent near-disappearance of the compound from the market, prompted the development of a number of alternative routes to TMZ and thus the imidazotetrazine core structure [47]. The first of these approaches was to use a masked methyl isocyanate equivalent (Scheme 6). Wang used commercially available isocyanoacetate **15** to synthesise an ester analogue of TMZ (**16**) by reacting with either Diazo-IC or AIC∙HCl. This compound was subsequently functionalized via acid **17** to carbonic anhydride **18**, converted to activated ester **19** and then subjected to Barton decarboxylation conditions to reveal the desired methyl group of TMZ [46].

*Reagents and conditions:* (i) Diazo-IC, DMSO, 25 °C (80%) *or* AIC∙HCl, pyridine, DMSO, 20 °C followed by NaNO_2_, 2 M HCl, 0 °C (71%); (ii) 5 M HCl, 45 °C; (iii) Me_2_CHCH_2_CO_2_Cl, *N*‑methylmorpholine; (iv) 2-mercaptopyridine-*N*-oxide, Et_3_N, −15 °C; (v) Bu_3_SnH, AIBN (cat.), DMF, hν (26%) [46].

**Scheme 7:**
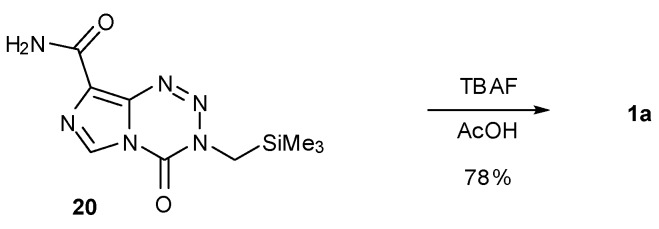
TMS deprotection to give TMZ [48].

Alternative masking of the methyl group can be achieved using silicon. TMS protected imidazotetrazine derivative **20** was synthesised in 33% yield from the appropriate isocyanate and diazo-IC; treatment with TBAF then furnished TMZ (Scheme 7) [48].

**Scheme 8:**
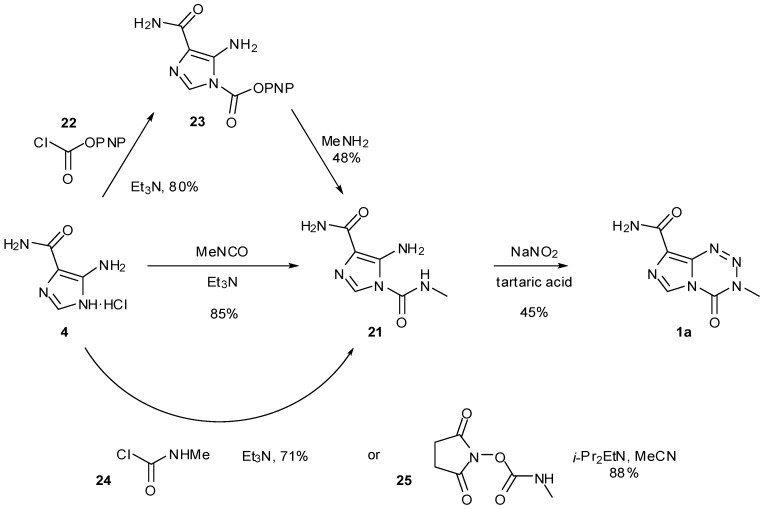
Routes to TMZ via urea **21** [47,49,50].

In addition to the use of alternative isocyanates, routes to imidazotetrazines avoiding the use of potentially-explosive diazo-IC were also explored (Scheme 8). The first of these started from AIC∙HCl but reversed the two steps of the original synthesis to proceed via urea **21**. Formation of ureas gives high yields however the subsequent imidazotetrazine cyclisation was found to be generally low yielding (30%–35% in the case of TMZ under standard conditions), with a significant amount of AHX seen as a by-product [47,49]. Alternative routes to urea **21** also obviated methyl isocyanate from this process. Reaction of AIC with *p*-nitrophenyl chloroformate (**22**) furnished carbamate **23** which was treated with methylamine to give the desired urea. A second modification allowed direct formation of the urea by carbamoylation of AIC with methylcarbamoyl chloride (**24**), whereas a related route by Turchetta *et al.* involved the use of *N*-succinimidyl-*N'*-methyl carbamate (**25**) as the carbamoylating agent [50]. Exploration of a variety of solvents, nitrosating agents and acids gave the optimum imidazotetrazine-forming conditions to TMZ as outlined in Scheme 8. A more recent patent gives alternative conditions for diazotisation using LiCl to give TMZ in up to 65% yield [51].

**Scheme 9:**
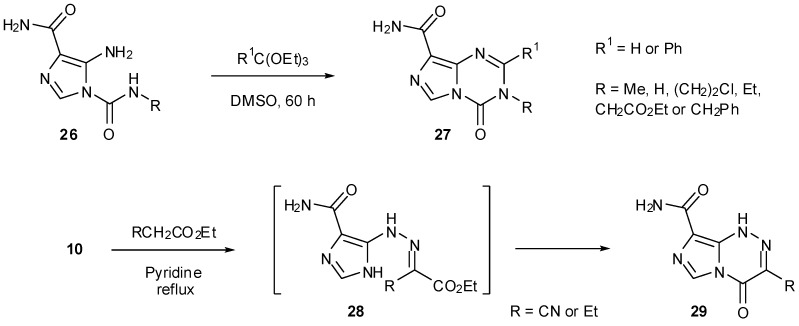
Synthesis of imidazotriazinones [25].

Treatment of ureas **26** with orthoformates rather than nitration gave rise to imidazotriazinones **27** (Scheme 9). Alternatively, reaction of diazo-IC with ethyl cyanoacetate or ethyl acetoacetate afforded regioisomeric imidazotriazinones **29** via unstable hydrazones **28** [25]. These types of heterocyclic structures, however, cannot give rise to biological activity in the same manner as the imidazotetrazines.

**Scheme 10:**
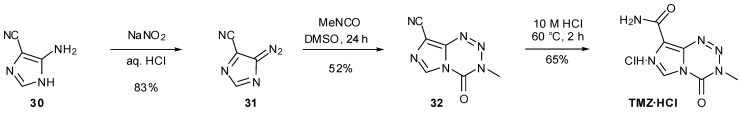
Synthesis of TMZ∙HCl from diazo-ICN (**31**) [47].

**Scheme 11:**
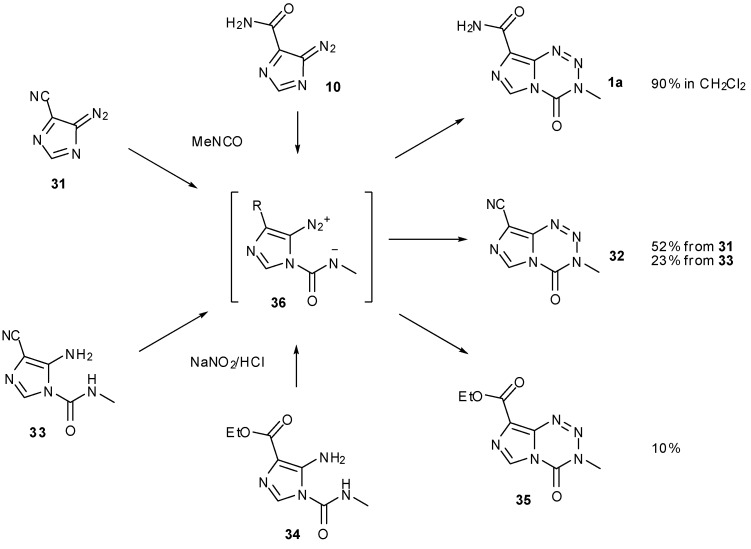
Summary of routes to amide alternatives [47].

A further alternative to bypass diazo-IC in imidazotetrazine synthesis involved switching the amide to a nitrile group (Scheme 10). It was hypothesised that removal of the amide functionality would suppress competing formation of unwanted side-product AHX. In fact, the yield from cyclisation of this nitrile compound **31** was lower than that obtained using diazo-IC. Following dehydration of AIC with phosphorus oxychloride, nitrile **30** was subjected to the standard diazotisation/cycloaddition conditions to give TMZ analogue **32** in 44% yield. Reversing these steps offered no improvement as formation of urea **33** was achieved in only 5% yield, resulting in a 1% yield of imidazotetrazine **32** over the two steps (Scheme 11).

The acid stability of TMZ allowed hydrolysis of the nitrile group with HCl, restoring the amide group and giving the desired product TMZ∙HCl in 65% yield for the final step [47]. The yield for this hydrolysis has since been improved by Etlin *et al*. (89%) by lowering the reaction temperature, along with a method for subsequent neutralisation of the hydrochloride salt [52].

No improvement to the cyclisation was achieved when the ethyl ester urea **34** was subjected to nitration conditions; in this case only a 10% yield of tetrazine **35** was observed (Scheme 11).

Neither nitrile nor ester intermediates **36** are able to undergo competing intramolecular cyclisation to give AHX-type compounds. The most likely explanation for the poor yields is therefore that such intermediates are sensitive to inductive effects; these side chains may be too electron withdrawing compared with the amide of TMZ, thus impeding cyclisation and allowing other degradation pathways to manifest. Wang *et al*. point out subtle reactivity patterns amongst these derivatized intermediates, highlighting the complexity in constructing and handling such ring systems [47].

**Scheme 12:**
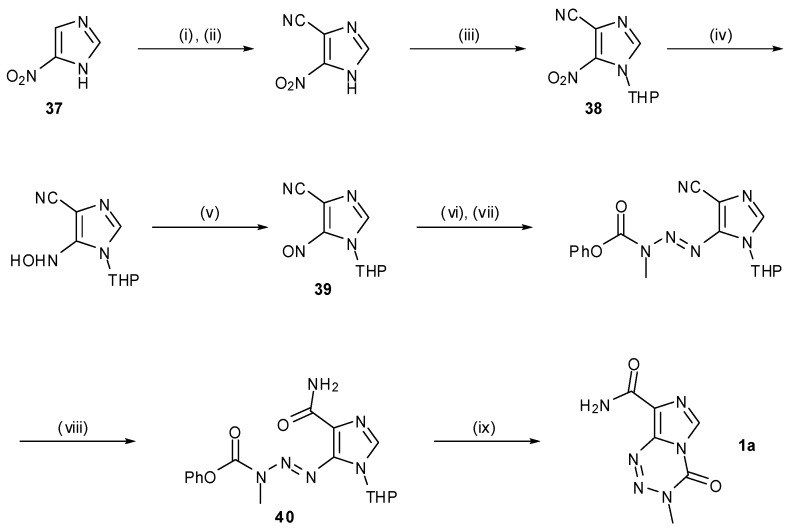
Wanner and Koomen’s route to TMZ.

A somewhat different approach to TMZ synthesis using an MTIC analogue was taken by Wanner and Koomen (Scheme 12) [53]. This route exploits a UV-activated double bond isomerisation of triazene **40**, which triggers spontaneous formation of TMZ. Beginning with 5-nitroimidazole (**37**), functionality is built up stepwise, culminating in condensation of **39** with phenylmethylcarbazate to provide the triazene moiety, followed by nitrile hydrolysis to furnish **40** prior to isomerisation. Whilst an elegant final step, the synthetic route to this key cyclisation precursor is comparatively lengthy *vis-à-vis* most other syntheses, with TMZ being made in an overall yield of 23% from imidazole **37**.

*Reagents and conditions:* (i) HNO_3_, Ac_2_O, 85%; (ii) KCN, NaHCO_3_, MeOH, H_2_O, rt, 62%; (iii) dihydropyran, *p*-TsOH, EtOAc, 96%; (iv) H_2_, 10% Pt/C, EtOAc, rt, 1 h; (v) NaIO_4_ in H_2_O, EtOAc, 0 °C, 30 min; (vi) H_2_N-N(Me)COOPh, CH_2_Cl_2_–AcOH (5:2), rt, 2 h; (vii) TFA–AcOH–MeOH (3:6:10), rt, 5 h, 76% from **38**; (viii) c. HCl–AcOH (2:1), 60–65 °C, 25 min, 74%; (ix) acetone–MeOH (2:1), 366 nm, rt, 1 h, 79% [53].

**Scheme 13:**
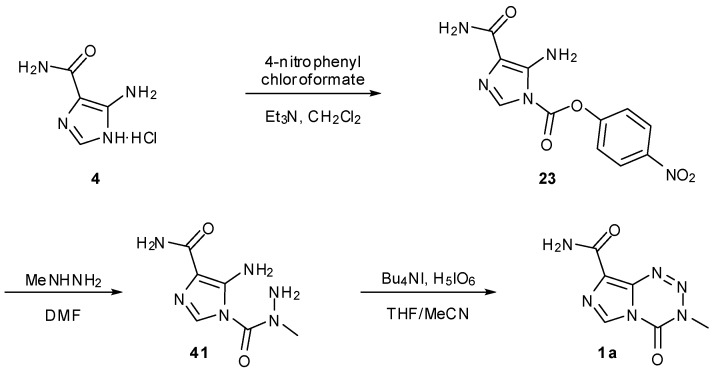
Schering-Plough industrial route to TMZ [54].

Schering-Plough Corporation has two patented procedures for the potential industrial scale synthesis of TMZ. The first of these avoids the handling of both diazo-IC and isocyanates but nevertheless has a familiar starting material. Following formation of carbamate **23** from AIC∙HCl; this activated intermediate was then treated with methyl hydrazine to give hydrazide **41**. Finally, periodic acid effected an unusual oxidative cyclisation to furnish TMZ (Scheme 13) [54].

**Scheme 14:**
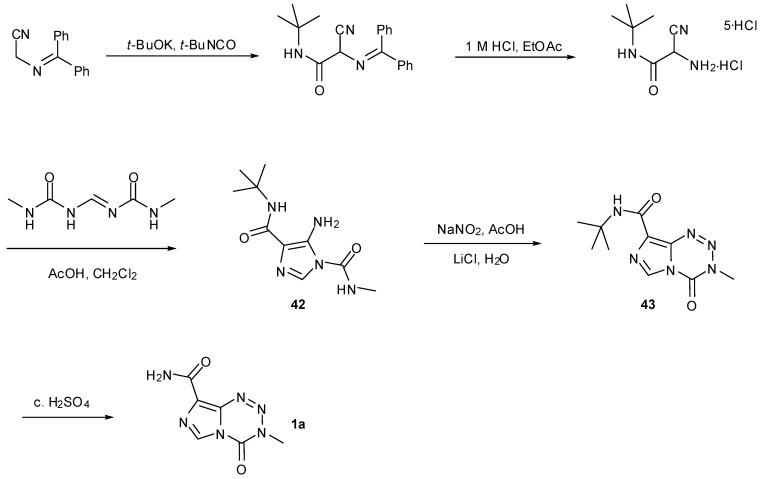
Schering-Plough alternative route to TMZ [55,56].

Their second and more cumbersome route requires construction of the imidazole ring during a lengthy route to urea **42**. Improved conditions for cyclisation afford imidazotetrazine **43** and acidic cleavage of the *tert*-butyl protecting group finally furnished TMZ (Scheme 14) [55,56].

**Scheme 15:**
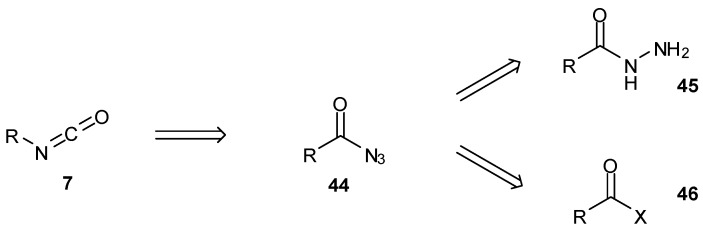
Common routes to isocyanates.

For small scale research purposes, coupling of diazo-IC with isocyanates in DMSO remains the method of choice for imidazotetrazine construction and offers unrivalled efficiency in terms of atom economy. Isocyanates **7** are most commonly formed via Curtius rearrangement of the corresponding azide **44**. The azides themselves can be constructed by subjecting the hydrazide **45** to diazotisation conditions, or by treatment of an activated carbonyl **46** with sodium azide or a carboxylate with dppa (diphenylphosphoryl azide) (Scheme 15). Isocyanates are notoriously difficult to handle and purify; Curtius rearrangements, as well as urea thermolysis, give access to isocyanates with high purity, a factor which has proved to be important in the synthesis of more elaborate imidazotetrazine compounds.

**Scheme 16:**
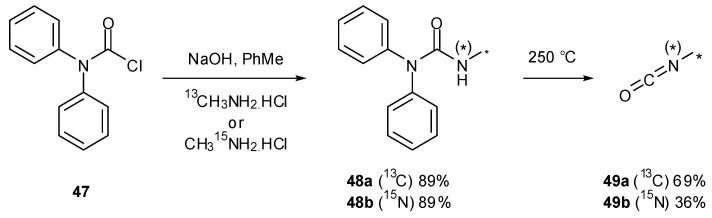
Urea pyrolysis to give labelled methylisocyanates [26].

**Scheme 17:**
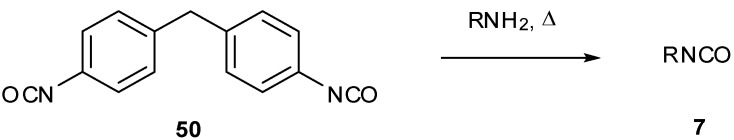
Thermolysis of 4,4'-methylenebis(phenylisocyanate) (**50**) [59].

Various studies have required the synthesis of isotopically labelled TMZ. The pyrolysis of ureas is an attractive method for the preparation of volatile isocyanates since pure products are collected via distillation [57]; this was exploited in the synthesis of isotopically labelled isocyanates in order to assemble ^13^C/^15^N-labelled TMZ. Reaction of diphenylcarbamoyl chloride (**47**) with either ^13^C- or ^15^N-methylamine gave ureas **48a** and **48b**, which under thermolysis created ^13^C- and ^15^N-methyl isocyanate (**49a** and **49b**) respectively (Scheme 16) [26]. This strategy has also been used for a number of other isocyanates during the formation of imidazotetrazines [25], including a process scale production of TMZ [58]. Access to isocyanates can also be achieved by the pyrolysis of 4,4'-methylenebis(phenylisocyanate) (**50**) in the presence of amines (Scheme 17) [59].

**Scheme 18:**
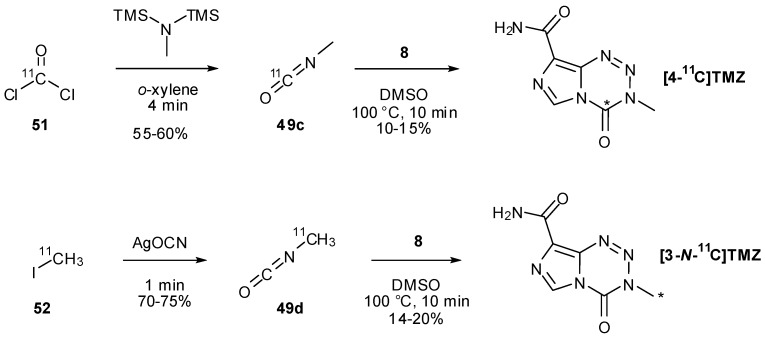
Alternative routes to isocyanates used in labelling studies [8].

Two other isocyanate-forming methods are demonstrated by Brown *et al*., who initially attempted to synthesise ^11^C-labelled TMZ exploiting the known intermediate in the TMZ activation pathway, MTIC (**2**), and coupling this with isotopically labelled phosgene [8]. As with previous efforts to effect this reaction [47], this was without success. They were able instead to utilise the radiolabelled phosgene (**51**) to rapidly obtain [^11^C-*carbonyl*]methyl isocyanate (**49c**) which was then used in a standard coupling reaction, gaining access to [4-^11^C-*carbonyl*]TMZ (Scheme 18).

An alternative synthetic route to the isocyanate employing [^11^C]iodomethane (**52**) and silver cyanate also allowed access to [3-*N*-^11^C-*methyl*]TMZ. Due to the short half-life (t_½_ = 20.33 min) of ^11^C, expeditious reactions are necessary in such radiosyntheses; Brown *et al*. found they were able to use elevated temperatures (100 °C for 10 min rather than room temperature for several hours) to significantly reduce reaction time in the coupling of isocyanates **49c/d** with diazo-IC (Scheme 18) [8].

**Figure 11:**
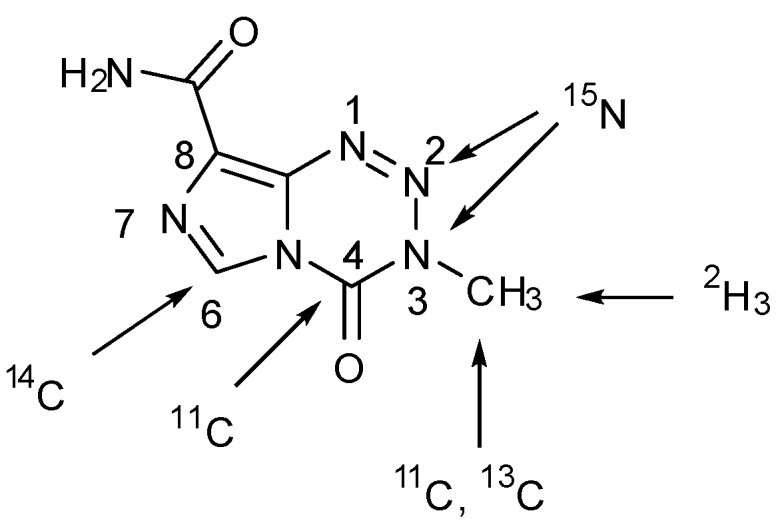
Summary of sites that have been isotopically labelled in TMZ [7,8,25,26,60,61,62,63].

2-^15^N-TMZ and [^2^H_3_-*methyl*]TMZ have also been synthesised by the standard method using [15N]diazo-IC (from Na^15^NO_2_) [26], and [^2^H_3_]methyl isocyanate respectively [25], and [6-^14^C-*imidazole*]TMZ has been used in several pharmacokinetic studies [7,60,61,62]. A summary of positions which have been labelled is shown in Figure 11.

## 7. Synthesis of Structural Analogues

Various TMZ analogues have been synthesized in efforts to find new, improved anti-cancer agents, using a range of methods to alter different parts of its structure.

### 7.1. Alternative Cores

It is clear from the mechanism of action that the tetrazinone ring is crucial for the activity of imidazotetrazine anticancer agents, which function via DNA alkylation; the importance of the imidazole on the other hand was initially less well understood. Pyrazolo analogues of MTZ and TMZ were initially made by Cheng *et al*., in order to generate agents with lower light sensitivity [64]. Whilst MTZ analogue **53** showed excellent antineoplastic activity against leukemia cell lines P388 and L1210, the corresponding TMZ analogue **54** was totally inactive [64]. It has, however, shown potency in the lymphoblastoid GM892A (MGMT−) cell line [24].

**Figure 12:**
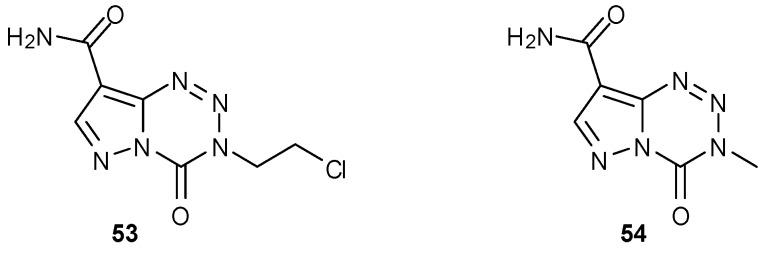
Pyrazolo analogues of MTZ and TMZ [64].

**Scheme 19:**
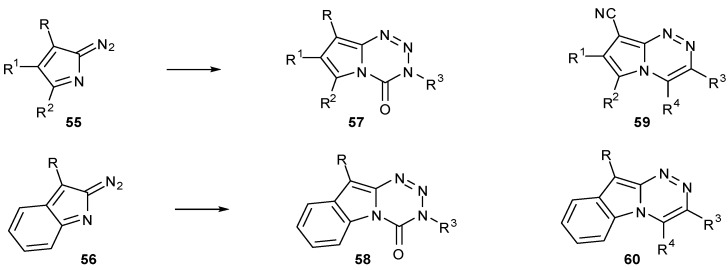
Heteroanalogues of imidazotetrazines [65,66].

A more thorough investigation into fused heterocycle effects has been made by the Cirrincione group, who have synthesised a range of alternative bicyclic ring structures related to the imidazotetrazines. Coupling isocyanates with diazopyrroles **55** and 2-diazoindoles **56** afforded pyrrolo[2,1-d][1,2,3,5]tetrazinones **57** and indolo[2,1-d][1,2,3,5]-tetrazinones **58** respectively (Scheme 19) [65,66]. Whereas the latter could be easily made using the standard procedure, the pyrrole series required more forcing conditions owing to reduced electrophilicity of the diazo group [65].

Some compounds showed modest activity in a range of cell lines in the NCI60 panel; of those in which growth inhibition was observed, GI_50_ values were generally in the 10–100 μM range. Indolo compounds **58** as a class were inactive (GI_50_ > 100 μM) [67], whereas pyrrolo compounds **57** showed a range of activity (GI_50_ > 100 μM to < 10 nM); the most potent compound in the series had a GI_50_ of <30 nM in a number of cell lines. QSAR trends and COMPARE analysis against the standard agents database indicated that the mode of action of pyrrolotetrazinones **57** differs from that of the imidazotetrazines [68,69]. Interestingly, some analogous triazines **59** and **60** also showed antiproliferative activity although as expected, COMPARE data highlighted that these too function by an alternative mechanism of action from members of the standard agents database [70,71].

**Scheme 20:**
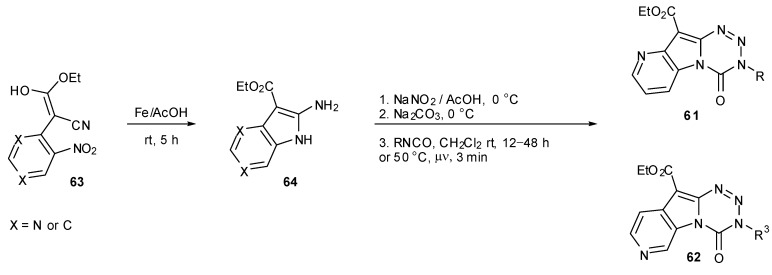
Synthesis of pyridopyrrolotetrazinones [72,73].

Diana and co-workers prepared two series of analogous pyridopyrrolotetrazinones **61** and **62**. Following construction of the pyrrolopyridine fragments **63** and **64**, standard diazotisation and coupling protocols gave the desired products. It is worthy of note that coupling with isocyanates was found to elicit higher yields when performed under microwave irradiation with greatly reduced reaction times (Scheme 20). Three of the compounds **62** showed growth inhibitory activity against a limited number of cell lines [72], but none of the compounds **61** tested in the NCI60 panel showed any significant activity [73].

**Figure 13:**
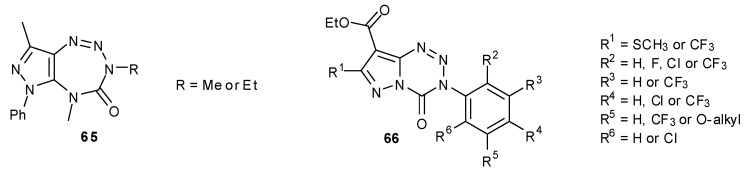
Unstable tetrazepines [74], and pyrazolotetrazinone herbicidal agents [76].

Two dihydropyrazolotetrazepines **65** were synthesised by Maggio and co-workers (Figure 13); however, these compounds were inherently unstable and thus their biological profiles could not be evaluated [74]. A number of pyrazolotetrazinones **66** has also been synthesized for use as herbicidal agents [75,76].

### 7.2. 6- and 8-Analogues

**Scheme 21:**
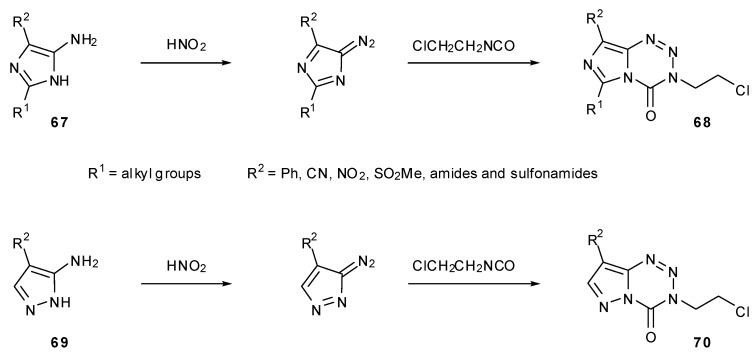
Synthesis of 6- and 8-analogues via substituted imidazoles [19].

Lunt and co-workers used a variety of alternatively substituted imidazoles **67** and pyrazoles **69** to create MTZ analogues **68** and **70** (Scheme 21) [19]. An assessment of the structure–activity relationship was first made by alteration of groups in the 6-position (R^1^). Several compounds were made with straight chain and branched alkyl groups of increasing size. Smaller substituents showed similar activity to MTZ but more bulky groups appeared to be less cytotoxic which is likely to be due to a steric barrier to hydrolysis.

A large range of analogues with different groups in the 8-position (R^2^), these mostly being amides and sulfonamides, exhibited a more complex structure–activity relationship. Increasing bulk of the amide substituent appeared to reduce cytotoxicity; the same trend was seen with sulfamoyl compounds but these as a class, along with 8-methylsulfonyl, showed enhanced potency compared with the amides. The 8-phenyl, 8-cyano and 8-nitro compounds tested showed no useful *in vivo* activity. Pyrazolotetrazines showed similar trends, but as well as being more complicated to synthesise, showed lower potency than the corresponding imidazotetrazines [19].

**Scheme 22:**
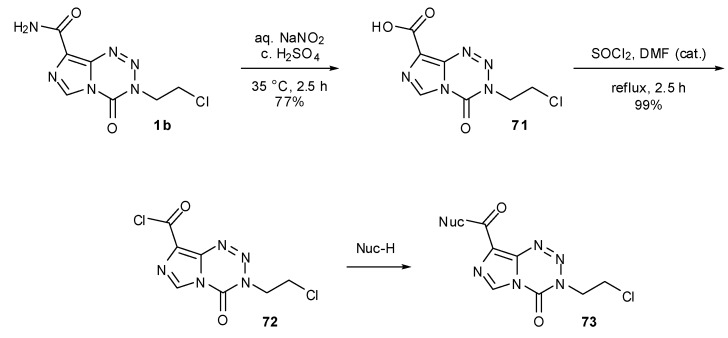
General synthesis of esters and amides via MTZ acid chloride [78].

Derivatisation of the 8-position of MTZ and TMZ can be achieved via treatment with nitrosylsulfuric acid (formed *in situ*) to hydrolse the amide moiety to carboxylic acid **71** (Scheme 22). Conversion to acid chloride **72** using refluxing thionyl chloride provides the necessary activation for selective reaction with nucleophiles, without destructive nucleophilic attack at the crucial endocyclic carbonyl. In this manner, large numbers of ester and amide analogues of MTZ **73** have been synthesised [77]. These compounds showed a broad range of activity in the TLX5 lymphoma cell line but no clear structure–activity relationship could be determined. This acid chloride approach was also utilised by Liu in the construction of a range of TMZ-based amides and esters. Esters were shown to have at least 60-fold higher water solubility than TMZ as well as more potent antitumour activity in HL-60 leukemia cell line [78].

**Scheme 23:**
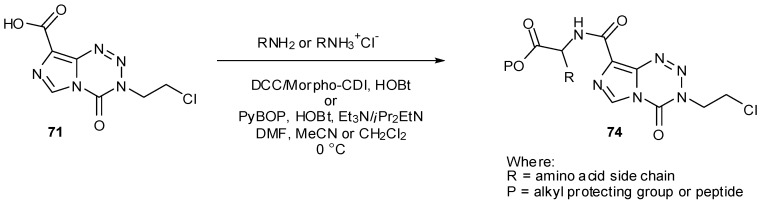
Synthesis of amides from MTZ carboxylic acid [45].

Esters and amides can also be achieved directly from carboxylic acid **71** by the use of alkyl halides or amines with coupling reagents such as DCC or phosphonium salts [79]. A number of conjugates **74** with amino acids on MTZ were prepared using this method, employing solid phase synthesis to generate longer chain DNA major-groove targeting peptidic analogues (Scheme 23) [45]. Care must of course be taken to avoid basic conditions in the presence of imidazotetrazines and with this in mind only acid labile groups were utilised for protection of amino acid side chain residues.

No significant change in aqueous stability was observed in this set of amide compounds and *in vitro* activity was not sufficiently improved to warrant further investigation. Imidazotetrazines conjugated to lexitropsins, a group of known distamycin-derived minor groove DNA binders were also synthesised by amide coupling. These conjugates were surprisingly water soluble but showed no enhanced activity. Regardless of the nature of the conjugate (major or minor groove targeting), alkylation was always seen at guanine-rich sequences in the major groove of DNA [45].

**Figure 14:**
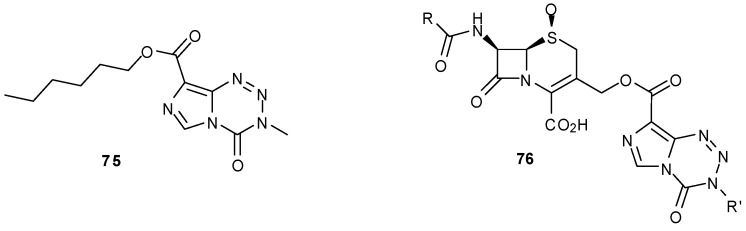
TMZ hexyl ester [80], and β-lactam antibiotics [81].

This coupling method was used in the synthesis of an assortment of temozolomide alkyl esters, thioesters and amides as prodrugs for topical application. Hexyl ester **75** was tested both *in vitro* and *in vivo* and showed improved dermal permeability compared with that of TMZ as well as demonstrating effective anti-melanoma properties (Figure 14) [80].

Wang also synthesized a number of amide/ester linked imidazotetrazines using PyBOP coupling in order to explore the dual action of MTZ/TMZ with cephalosporin antibiotic agents, with an aim to increase the spectrum of activity of the parent antibiotics (Figure 14) [81]. However, dual action β-lactam agents **76** were not as effective as ampicillin against most bacterial strains and little synergistic activity was seen.

**Scheme 24:**
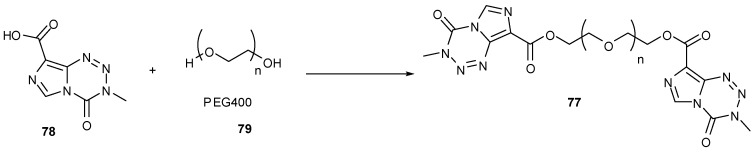
Glycol linker dimer [82].

An ethylene glycol linked dimer of TMZ **77** (Scheme 24), synthesised from acid **78** and PEG400 (**79**), when administered intravenously showed equal efficacy against glioma cells *in vivo* compared with oral dosing of TMZ. In addition, a mouse xenograft model showed tumour growth inhibition comparable to that exhibited by TMZ [82].

**Scheme 25:**
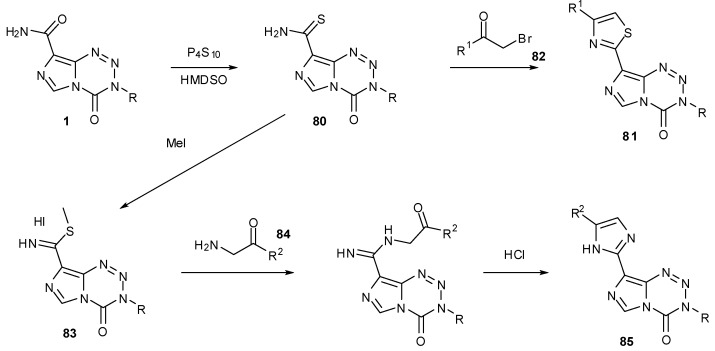
Formation of thiazoles and imidazoles [83].

An alternative way to derivatise the 8-position is to convert the carboxamide to a thioamide. Hummersone and co-workers used phosphorus pentasulfide and hexamethyldisiloxane to elaborate on the 8-position. These thioamides **80** can then be further derivatised to thiazoles **81** by condensation with an appropriate α-bromo ketone **82**. Alternatively, conversion of thioamides to carbimidothioates **83** then reaction with aminoketones **84** gives access to imidazoles **85** (Scheme 25) [83].

**Scheme 26:**
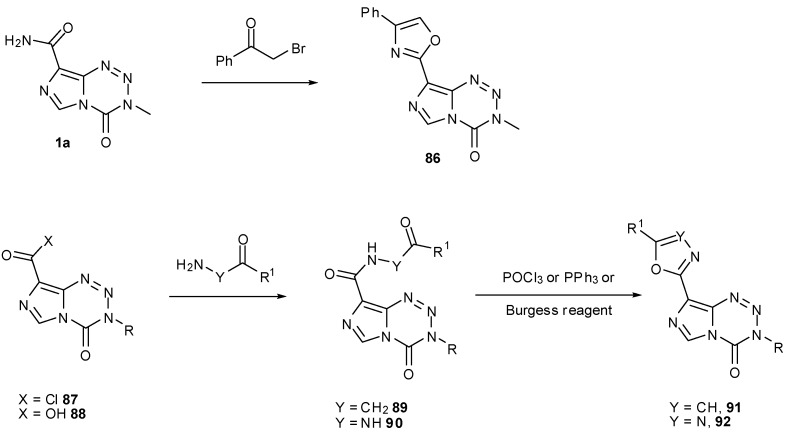
Construction of oxazoles and oxadiazoles [83].

Oxazoles **86** can similarly be synthesised by treatment of carboxamides, e.g., **1a** with α-bromo ketones. Alternatively, reaction of acid chloride **87** with an appropriate amino ketone to form keto amides of the form **89** followed by ring closure and dehydration gives regioisomeric oxazoles **91**. In a similar manner, reaction of carboxylic acids **88** with hydrazides allows construction of oxadiazoles **92** via **90** (Scheme 26). A large number of these 8-substituted compounds was found to have effective biological activity, even in cell lines that have active MGMT; many compounds showed GI_50_ values <45 μM (compared to 45.6 μM for TMZ) in SNB19V (MGMT−) and <70 μM (compared to 526.3 μM for TMZ) in SNB19M (MGMT+) [83].

### 7.3. 3-Analogues

**Scheme 27:**
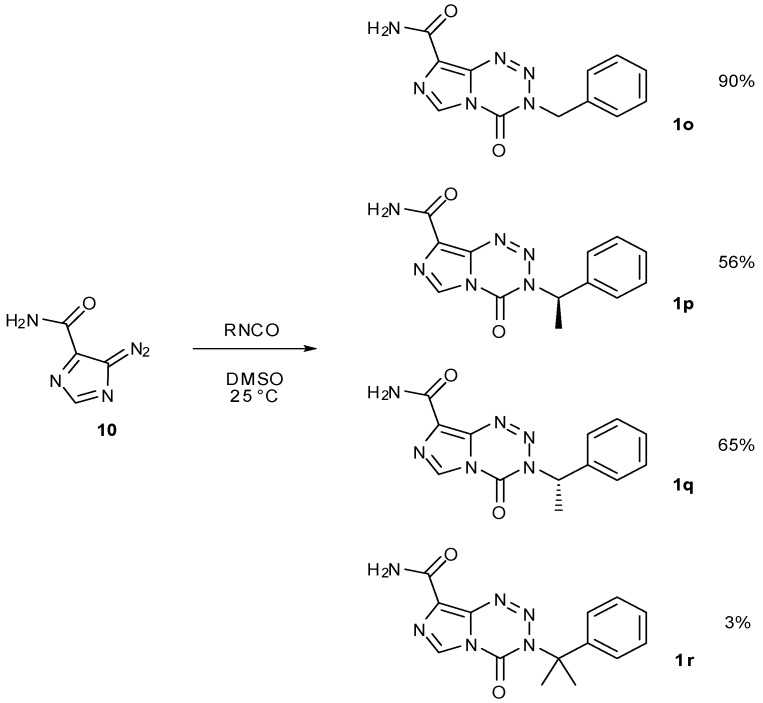
Example of steric effects on the diazo-IC–isocyanate coupling reaction [43,84,85].

The 3-substituent in imidazotetrazine compounds is crucial as this is the group that controls reactivity of the imidazotetrazine and will also be delivered and subsequently bonded to DNA. A large number of 3-*N*-substituted imidazotetrazines has been synthesised, bearing a variety of functionalised alkyl groups as well as alkyl-linked aromatics and heterocycles [25,84,85]. These have been constructed mostly using isocyanate methods. The effects of steric hindrance on this coupling reaction with diazo-IC (**8**) are evident; benzyl isocyanate gives 90% yield of **1o** [43], whereas (*R*)- and (*S*)-1-phenylethyl isocyanate after 16 h afford imidazotetrazines **1p** and **1q** in 56% and 65% yield respectively. Somewhat surprisingly, reaction with, α-dimethylbenzyl isocyanate does furnish the *gem-*dimethyl analogue **1r**, albeit it in a yield of only 3% after 16 h reaction time (Scheme 27) [84,85].

**Scheme 28:**
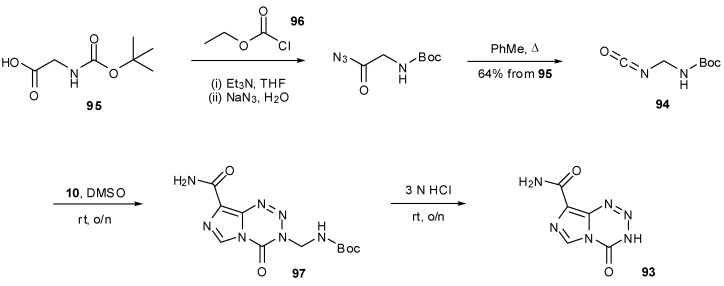
Synthesis of nortemozolomide [86,87].

A more recent development in imidazotetrazine synthesis employs the intermediate, nortemozolomide (**93**). The route to **93** uses diazo-IC (**10**) and glycine-derived isocyanate **94**, itself formed via azide displacement of the anhydride resulting from reaction with Boc-glycine (**95**) and ethyl chloroformate (**96**), and subsequent Curtius rearrangement. Following imidazotetrazine formation, Boc-deprotection of **96** leads to spontaneous elimination of formaldehyde imine, thus resulting in formation of nortemozolomide (Scheme 28) [86,87]. This imidazotetrazine compound opens up scope for not only the synthesis of TMZ but also creates opportunities for alternative routes to 3-*N*-substituted analogues.

**Scheme 29:**
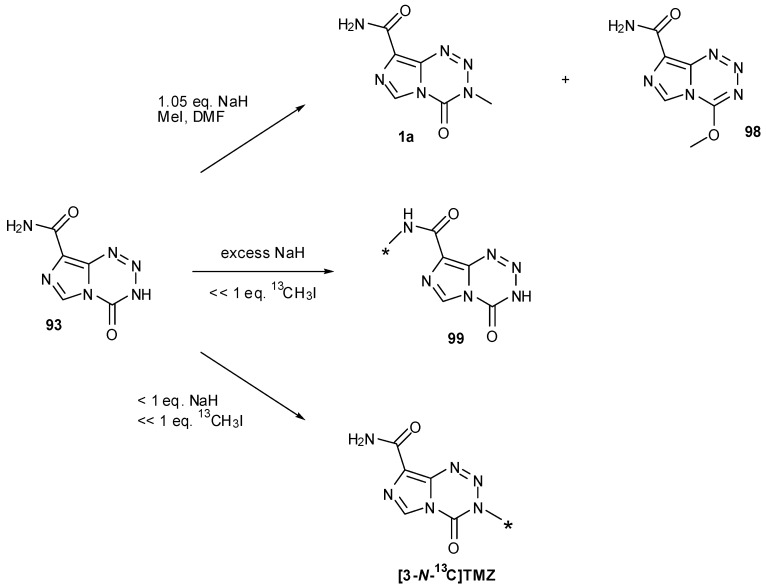
Methylation of nortemozolomide [63,87].

Methylation of **93** to give TMZ was achieved by Cousin *et al*., in 61% yield by deprotonation with sodium hydride and subsequent quenching of the anion with an excess of methyl iodide in DMF. An *O*-methylated side-product was also detected, suspected to be **98**; alteration of base had little effect on the outcome of the reaction but less side-product was obtained when the reaction was performed at lower temperatures (−20 °C) [87]. During the course of their [^13^C]-and [^11^C]-labelled TMZ syntheses, Moseley and co-workers determined that sodium hydride was the optimum base for methylation of nortemozolomide in order to reduce 8-*N*-methylation. However, they observed that the reaction is sensitive to the amount of base and found that with [^13^C]-methyl iodide, excess NaH resulted in exclusive 8-*N*-methylation to give **99** and that substoichiometric quantities favoured 3-*N*-methylation (Scheme 29) [63].

**Scheme 30:**
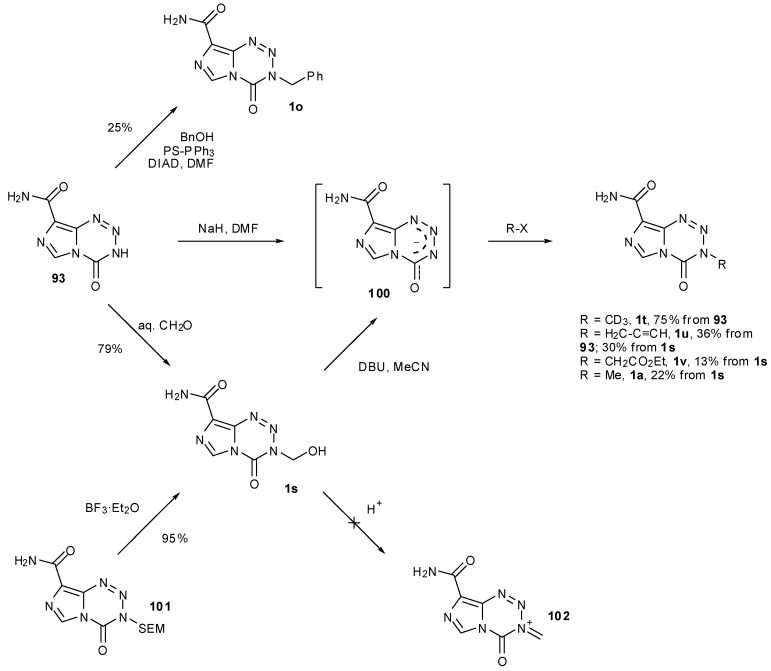
Alkylation of nortemozolomide [84,87].

The derivatization of **93** using a number of alkylating agents was also investigated. The scope was shown to be limited, as alkyl halides with bulky or electron withdrawing substituents failed to yield the desired products. The benzyl derivative **1o** was obtained under Mitsunobu conditions whereas hydroxymethyl compound **1s** was formed from simple treatment of **93** with formaldehyde (Scheme 30). This alcohol itself was next probed as a gateway to diversification. Exposure to acid gave only ring decomposition products rather than the anticipated iminium species **102** which it was envisioned could have provided access to ethers and thioethers. On the other hand, treatment of **1s** with DBU and trapping with electrophiles provided alternative, if less efficient conditions for access to simple alkylated systems, presumably via anion **100**. Alcohol **1s** is also accessible in high yield from SEM protected nortemozolomide **101** via BF_3_ etherate deprotection, albeit with some BF_3_ contamination [84,87].

## 8. Design of MGMT/MMR-Independent Anti-Cancer Agents

The principal constraints on TMZ efficacy are the dependence on MMR and resistance caused by MGMT, the result of which is that the number of tumours able to respond to TMZ therapy is limited; current efforts in the design of new analogues seek to bypass these limitations.

Although much attention has been paid to the modification of the 8-substituent and exploring the effects of targeting ligands attached at this position, it is actually the 3-subsituent that generates the pharmacological activity of the imidazotetrazines. Recent efforts to modify tumour response to TMZ-type drugs have focused on this position. Key to rationally altering the 3-substituent and the biological activity is a full appreciation of the role of the 3-substituent in the imidazotetrazine mechanism of activation and reactivity.

**Figure 15:**
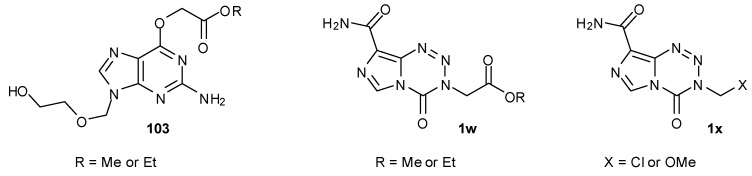
Compounds resistant to MGMT effects [85,88,89].

One approach towards achieving this is to modify the 3-substituent of the imidazotetrazine such that the group transferred to DNA G*O*6 sites is a poor substrate for MGMT and thus is not susceptible to recognition or DNA repair by the protein. A family of alkylated guanine molecules **103** with polar or ionisable *O*6 substituents has been synthesised and demonstrated resistance to MGMT cleavage (Figure 15) [88]. A number of imidazotetrazine 3-*N*-derivatives bearing such types of substituents (e.g., **1w** and **1x**) has demonstrated MGMT- and MMR-independent activity; several cell lines that are resistant to TMZ, whether due to high levels of MGMT or MMR mutation/deficiency, have shown sensitivity to these new compounds [85,89].

Another approach is to subtly shift the mode of action, which has been achieved by embracing a neighbouring group participation (NGP) mechanism to alter the behaviour of the active species resulting from prodrug release.

### 8.1. Introduction to NGP Analogues

**Scheme 31:**
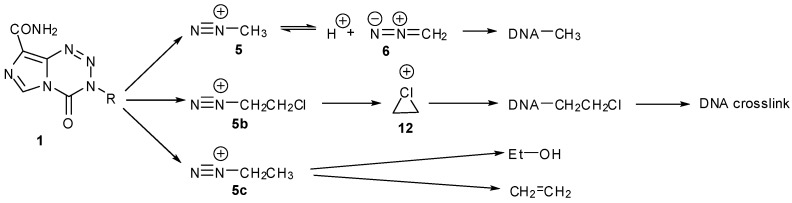
Summary of alkyldiazonium ions derived from early imidazotetrazines.

Earlier, the aqueous chemistry of the early imidazotetrazines was discussed (*vide supra*, Section 3), summarised in Scheme 31. The important lesson here is that for a drug to elicit a biological response, fine tuning of the latent electrophile is required. Firstly so that unproductive side-reactions, such as inactivation by elimination, hydrolysis or rearrangement, are disfavoured. More subtly, it is also important that the ultimate electrophile has sufficient aqueous lifetime to locate an appropriate reaction site on DNA. Stabilisation of diazonium ions **5**, **5b**, by either equilibration with diazomethane (**6**) or involvement of the neighbouring chlorine atom to form a chloronium intermediate **12**, appears adequate to ensure useful pharmacological activity. In contrast, ethyl diazonium (**5c**) is not stabilised by equilibration with the diazo form but is susceptible to elimination which provides a “dead end” to the sequence of reactive intermediates.

**Figure 16:**
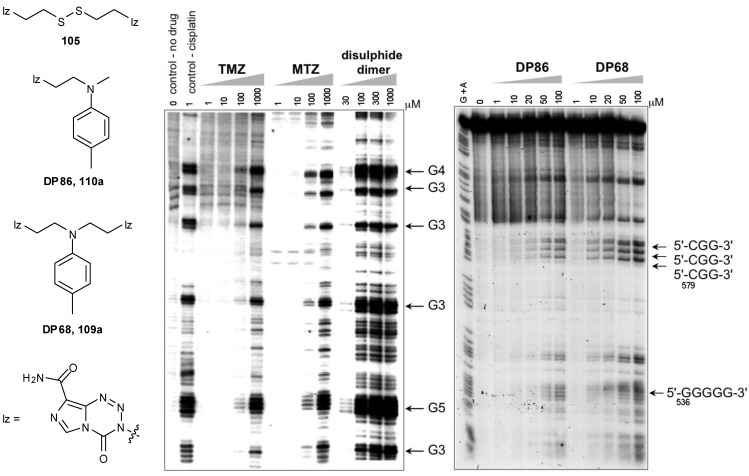
(**Left**) Comparison of reaction of TMZ, MTZ and a disulphide-linked dimer **105** with DNA showing the enhanced reactivity of the episulphonium prodrug, sites of DNA alkylation detected by the Taq DNA polymerase stop method (adapted from reference [90]); (**Right**) Reaction of Aziridinium Prodrugs (DP86 and DP68, **107a** and **108a**) showing the increased reactivity of the bisimidazotetrazine. Sites of G*N*7 alkylation on the upper strand of pBR322 DNA detected by the piperidine cleavage method. Arrows indicate the position and sequence context of the alkylated guanines. Adapted from reference [93].

**Scheme 32:**
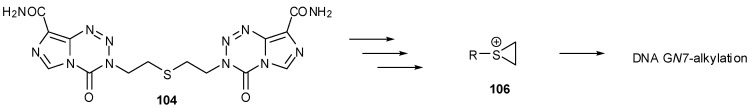
Generation of episulfonium ions from a sulfur-linker dimer [90].

Control of the reactive intermediates has been achieved using neighbouring group participation mechanisms, inspired in part by the chloronium ion of MTZ and the aziridinium intermediates generated by nitrogen mustard prodrugs. Moreover, bisimidazotetrazines were designed that combined two imidazotetrazines to form potential DNA crosslinking agents. The first examples used sulfur as the heteroatom, e.g., **104**, **105** and led to both effective DNA alkylation (Figure 16), and activity in the NCI60 panel [90]. The good reactivity with DNA was consistent with the generation of episulfonium ions **106** which are relatively long-lived intermediates in aqueous systems (Scheme 32) [91]. Whilst demonstrating the validity of the design principles, further development of the thioether and disulphide linked compounds was disfavoured as NCI COMPARE analysis indicated close relationships with existing agents such as thiotepa, chlorambucil and uracil mustard (*p* > 0.8) [92].

Potential aziridinium ion precursors were attractive as these are reactive intermediates of proven clinical utility, being found widely in, or generated by, synthetic and natural product anti-tumour drugs and prodrugs so may be expected empirically to display suitable pharmacokinetics.

### 8.2. Synthesis of Novel N-Linked Imidazotetrazine Dimers

**Figure 17:**
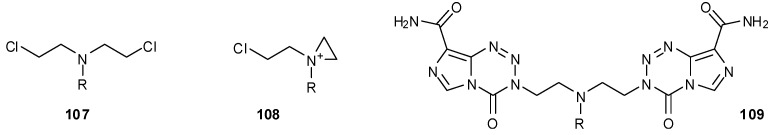
Nitrogen mustards, resulting aziridinium ions and novel imidazotetrazine dimers.

This new class of compounds employed nitrogen as the heteroatom controlling the cascade of reactive intermediates in a manner analogous to the nitrogen mustard drugs **107** (Figure 17). These anticancer agents are DNA cross-linkers which react via aziridinium ions **108**; these are ring opened during nucleophilic substitution reactions with groups on DNA, principally *N*7 of guanine residues.

These novel *N*-linked dimers **109** were rationally designed as acid-stable precursors to aziridinium ions, to combine the useful pharmacokinetic properties of the imidazotetrazines with the proven clinical utility of DNA cross-linkers. By exploiting preferential G*N*7 alkylation (70% for TMZ), as opposed to the mere 5% alkylation at G*O*6, an efficiency gain was sought; moreover, the requirements for MMR and absence of MGMT may be negated.

**Scheme 33:**
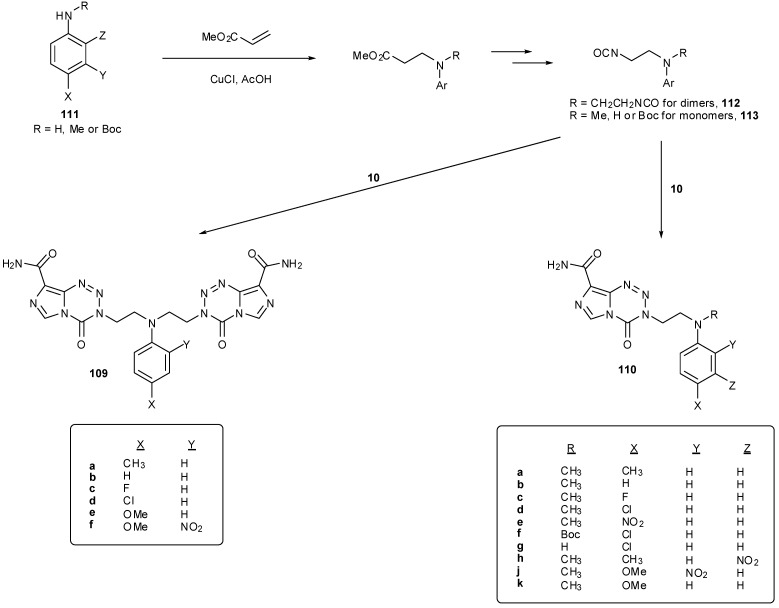
Synthesis of *N*-linked dimers and momomers from diisocyanates and isocyanates [93,94,95].

Synthesis of these dimer compounds **109** was via diisocyanates, an approach that had already proved successful in the construction of dimeric imidazotetrazines [25,45]. A family of monomeric imidazotetrazines **110** with aminoethyl side chains has been similarly prepared. The required substrates were obtained from conjugate addition of primary and secondary anilines **111** with methyl acrylate followed by functional group interconversion to give diisocyanates **112** and isocyanates **113** respectively (Scheme 33) [93,94,95].

**Scheme 34:**
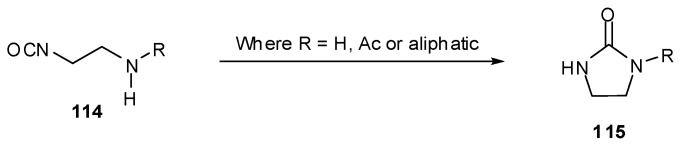
Cyclisation of β-aminoisocyanates.

Imidazotetrazine rings cannot be closed in the presence of tertiary amines unless the nucleophilic centre is deactivated. This can be achieved through the use of anilines or by *N*-Boc protection; these techniques also serve the purpose of preventing undesired cyclisation of β-aminoisocyanates **114** to cyclic ureas **115** (Scheme 34) [95]. It is worthy of note that an acetate group is insufficiently electron withdrawing for *N*-deactivation. Little difference in reaction yield was seen between secondary and tertiary anilines and Boc-aniline isocyanates **113** to give **110**.

### 8.3. Properties and Activity of N-Linked Compounds

Both classes of new compound show similar hydrolysis kinetics to TMZ, with almost identical half-lives at pH 7.4 and pH 8.0; acid stability is still robust although not as pronounced as for TMZ [93].

**Scheme 35:**
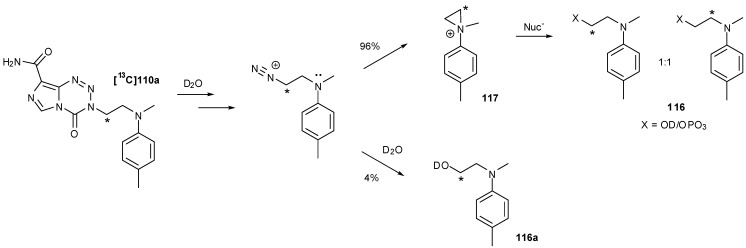
Aziridinium release [93].

An isotopically labelled version of monomer **110a** was prepared for mechanism studies using enriched ^13^C(2)-acrylate. On exposure to D_2_O phosphate buffer (pD 7.8), a mixture of phosphate and alcohol (hydrolysis) products **116 **was observed. From ^13^C-NMR studies it could be seen that there was a 4% excess of the alcohol **116a** resulting from direct hydrolysis, yet amongst the remaining 96% there was a complete scrambling of the labelled centre, thus proving the formation of an aziridine intermediate **117** (Scheme 35) [93].

Reaction of **109a** and **110a** with DNA in a piperidine cleavage assay showed that alkylation sites were identical to those obtained with melphalan. In this assay, dimeric compound **109a** was more reactive than monomer **110a **and showed reactivity with DNA equivalent to melphalan (Figure 16) [93]; in whole cells, dimer **109a **showed DNA crosslinking activity equivalent to melphalan [96].

Both dimers **109** and monomers **110** showed promising *in vitro* anti-cancer activity. Preliminary screening was undertaken against an MGMT and MMR-proficient cell line (A2780) and the associated MMR-deficient A2780-cp70; MGMT was inactivated with PaTrin2 in order to evaluate both MMR and MGMT dependence. Pleasingly, members of both classes of compound showed anti-proliferative activity, regardless of MGMT and MMR status of the cell lines tested. The monofunctional agents showed activity equivalent to MTZ, whereas the bifunctional agents showed significantly greater potency than MTZ [95].

**Figure 18:**
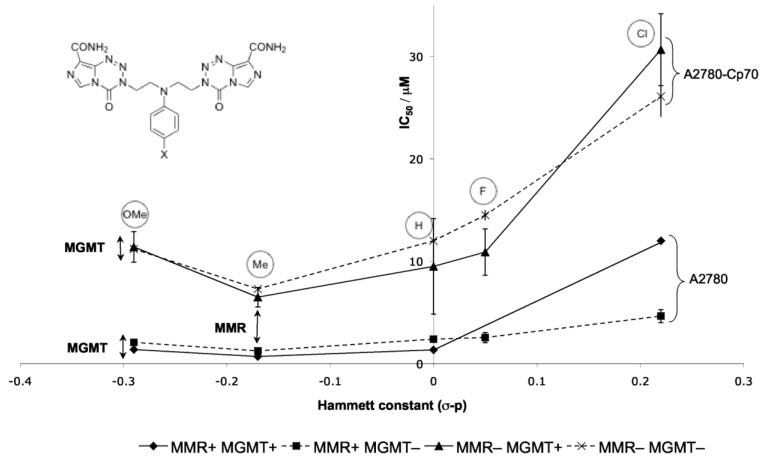
Effect of electron-withdrawing capabilities of substituent X on the activity of aniline dimers **109** [95]. The differences between sets of lines indicate the limited MMR/MGMT effects.

Probing more deeply into the *in vitro* SAR of the aniline dimers **109** showed a trend in Hammett constant *vs.* IC_50_; with the exception of the 4-methoxy derivative, the more electron-donating the aniline substituent, the better the activity (Figure 18). This shows a clear link between electronics of the 4-subtituent and pharmacological activity. This can be rationalised by electron donation providing enhanced formation and stability of the aziridinium alkylating agent; the anomalous methoxy group on the other hand potentially increases basicity too much so that appreciable protonation of the aniline occurs, thus reducing nucleophilicity.

**Table 2:** Results of Matrix COMPARE (P values) for NCEs **109c**–**e**, **110d**, with selected standard agents. DTIC, 5-(3,3-dimethyl-1-triazeno)imidazole-4-carboxamide (**3**); MEL, melphalan; CHB, chlorambucil; BCNU, 1,3-bis(2-chloroethyl)-1-nitrosourea; CCNU, *N*-(2-chloroethyl)-*N'*-cyclohexyl-*N*-nitrosourea; CP, cisplatin; Data from reference [95].

	109c	109d	109e	110d	MTZ	DTIC	MEL	CHB	BCNU	CCNU	CP
**109c**	1	0.92	0.92	0.83	0.35	0.72	0.4	0.33	0.32	0.06	0.32
**109d**	0.92	1	0.81	0.79	0.38	0.64	0.36	0.29	0.27	0.05	0.35
**109e**	0.92	0.81	1	0.77	0.46	0.71	0.59	0.53	0.45	0.10	0.42
**110d**	0.83	0.79	0.77	1	0.42	0.62	0.35	0.33	0.25	0.08	0.27

Moreover, NCI screening data confirmed the lack of relationship between MGMT, MMR and efficacy. Matrix COMPARE analysis showed the distinctiveness of these compounds against other aziridinium/diazonium-releasing or crosslinking agents such as MTZ, the nitrosoureas, nitrogen mustards and cisplatin (Table 2).

## 9. Conclusions

The imidazotetrazine prodrugs are an interesting class of pharmaceutically active compounds. The original synthetic strategy towards the bicyclic imidazotetrazine core employed coupling of diazo-IC with isocyanates; the driving forces for alternative syntheses were based around the stigma and lack of availability of methyl isocyanate as well as avoidance of potentially explosive diazo-IC. Ultimately, however, none of the procedures have bettered the initial route and this remains the preferred method for laboratory scale synthesis.

Ring opening follows two mechanisms, the retro-cycloaddition and hydrolytic prodrug activation mechanism. The latter occurs in two steps: hydrolysis of the imidazotetrazine ring followed by acid-mediated release of alkyl diazonium ions. The mechanism and kinetics of this prodrug activation and thus the circulating half-life are determined by the 3-substituent of the imidazotetrazine. The nature of this group plays a huge role as it also ultimately determines the biological response. In addition to controlling prodrug activation, controlling the reactivity of the released electrophile is of vital importance to allow reaction with DNA.

Response of a tumour to TMZ treatment is dependent on two factors: absence of MGMT, the protein which results in inherent resistance, and presence of MMR, the actions of which ultimately lead to cell death. In addition to these constraints, it is only a minor proportion of DNA alkylation (5% on G*O*6) which actually results in the anti-tumour response. Recent efforts to combat MGMT resistance have focused on compounds which create adducts unrecognisable by MGMT or those which instead are able to elicit a pharmacological response from G*N*7 alkylation. Moreover, the need for a non-mutagentic TMZ derivative is urgent, now that the molecular details of the relationship between TMZ-induced mutation and the evolution of aggressive, drug resistant tumour regrowth, following initial good response to therapy, has been established [97].
